# Scenario-based Kaya identity analysis for city-level carbon dioxide emissions

**DOI:** 10.1371/journal.pone.0329937

**Published:** 2025-08-08

**Authors:** Junyou Liu, Bohong Zheng

**Affiliations:** School of Architecture and Art, Central South University, Changsha, Hunan, China; Government of Nova Scotia, CANADA

## Abstract

Many countries worldwide have committed to reduce their carbon dioxide emissions in response to climate change. In China, cities are striving to achieve their 2030 peak carbon emission targets. In this study, we developed a scenario-based Kaya identity analysis methodology to explore the notable uncertainty inherent in future carbon dioxide emissions. Using the case of Changning City, Hunan province, China, we found that under the business-as-usual scenario, the city’s carbon dioxide emissions of 3,839.1 thousand tons in 2022 are projected to reach 4,674.3 thousand tons in 2031. Changning is unlikely to achieve its carbon peak target. In a more challenging future scenario of rapid economic growth, carbon dioxide emissions are expected to rise from 3,839.1 thousand tons in 2022–5,447 thousand tons in 2031. Under the environmentalist scenario, Changning could achieve its carbon peak target before 2030 (carbon dioxide emissions would peak at 3,922 thousand tons in 2028). Under a slowed economic development scenario, Changning could also achieve a carbon peak in 2028 (carbon dioxide emissions would peak at 3,980.2 thousand tons in 2028). However, without sufficient energy-saving and emissions-reduction measures, annual increases in carbon dioxide emissions are likely to resurge during periods of rapid economic development.

## 1. Introduction

Carbon dioxide (CO_2_) is among the most important greenhouse gases and a major contributor to global warming. In 2021, the average global temperature was approximately 1.1°C higher than the pre-industrial level [1]. Climate change not only makes several cities intolerably hot in summer but also melts glaciers, with many cities at the risk of being submerged [2]. In 2015, 196 parties to the United Nations Paris Agreement committed to limit global warming to well below 2°C from the pre-industrial level, while aspiring to limit warming to 1.5°C [1–3].

As one of the world’s largest developing countries, China is among the top three greenhouse gas-emitting countries [4]. In recent decades, China has made significant efforts to reduce its CO_2_ emissions and alleviate the heat island effect. In 2020, the Central Committee of the Communist Party of China led by President Jinping Xi proposed a dual-carbon strategy that China’s total CO_2_ emissions will reach a historical peak by 2030 and not increase thereafter. By 2060, it is projected that China will achieve a balance between anthropogenic emission sources and absorption through carbon sinks such as afforestation and carbon capture and storage (CCS) technologies [5]. Thus, all levels of government in China are committed to achieve these dual-carbon strategic goals.

Uncertainties related to factors such as future urbanization rates, gross domestic product (GDP) per capita, research and development (R&D) intensity, energy structure, population, and energy intensity (energy consumption per unit of GDP), make it challenging at all levels of government to achieve the carbon peak goal within their jurisdiction, including the uncertainty of timing [6,7]. To achieve the strategic goal of carbon peak by 2030, it is critical to address the uncertainty about the timing of the carbon peak and propose optimization strategies.

## 2. Literature review

Several studies have used different methods to estimate future CO_2_ emissions. For example, Wei et al. [8] predicted CO_2_ emissions in China’s Henan Province using the Tapio method and Stochastic Impacts by Regression on Population, Affluence, and Technology (STIRPAT) model. Köne and Büke [9] explored CO_2_ emissions under different emission scenarios based on the Intergovernmental Panel on Climate Change (IPCC) guidelines, considering the energy mix, energy intensity, carbon intensity (CO_2_ emissions per unit of GDP), and population. Wang et al. [10] used the IPCC regional emissions accounting method to calculate CO_2_ emissions from 15 cities in Sichuan Province, China. Sun et al. [11] used a Long-range Energy Alternatives Planning (LEAP) software system to simulate CO_2_ emissions in China’s Suzhou City under multiple scenarios.

The Kaya identity explores CO_2_ emissions, relying primarily on GDP per capita, population, energy intensity, and carbon intensity. Scenario planning is beneficial for exploring future uncertainty. Previous studies used it to explore future uncertainty and calculate the amount of CO_2_ emissions in different scenarios based on the Kaya identity. Liu et al. [12] conducted a multi-scenario simulation analysis using the Kaya identity to model energy consumption and related emissions in China and quantitatively analyzed possible future scenarios for a several factors (e.g., population, GDP, industrial structure, energy intensity, residential income, household energy consumption, and energy consumption). They noted that it is necessary to enhance policies beyond 2030 to achieve the carbon neutrality pledges. Liu et al. [12] considered two to three scenarios for the baseline, acceleration, and low speed for each factor. A combination of several factors can result in a considerable number of scenarios. However,. only a small number of CO_2_ emissions scenarios were produced. Zhao et al. [13] used the Kaya identity to explore CO_2_ emissions in the Beijing-Tianjing-Heibei region of China under various scenarios. Under the baseline scenario, they found that the region would not achieve its carbon emissions peak before 2030. Hence, further regulation of energy taxes, technological investment, and stricter policy restrictions are necessary to ensure that the carbon peak target can be achieved by 2030 under rapid economic development. Zhao et al. [13] built four scenarios (rough, intermediate, transformation, and sustainable development) using six main factors (GDP growth rate, renewable energy tax, CO_2_ reduction policies, population growth rate, proportion of R&D, and proportion of environmental protection investment). They also used a system dynamics model to explore future uncertainties. Considering the example of Shenzhen, Wang and Xie [14] used decoupling theory and Kaya identity to explore future scenarios for CO_2_ emissions in the tourism economy development. They found that the relevant decoupling scenario was most conducive to the development of the tourism industry, based on a scenario that focused on one’s own reality between the two benchmarks of advanced domestic and international levels and historical development trends. This scenario gradually changes the current economic development mode, maintaining rapid gross domestic product (GDP) growth in the tourism industry and achieving a significant upsurge in energy productivity compared to historical trends, while significantly improving resource consumption and CO_2_ emissions.

Some studies have used scenarios and the Kaya identity to explore future CO_2_ emissions; however, most studies have conducted these two steps separately. Future CO_2_ emission scenarios were developed and calculated under different scenarios based on the Kaya identity.

## 3. Methodology

**Fig 1:**
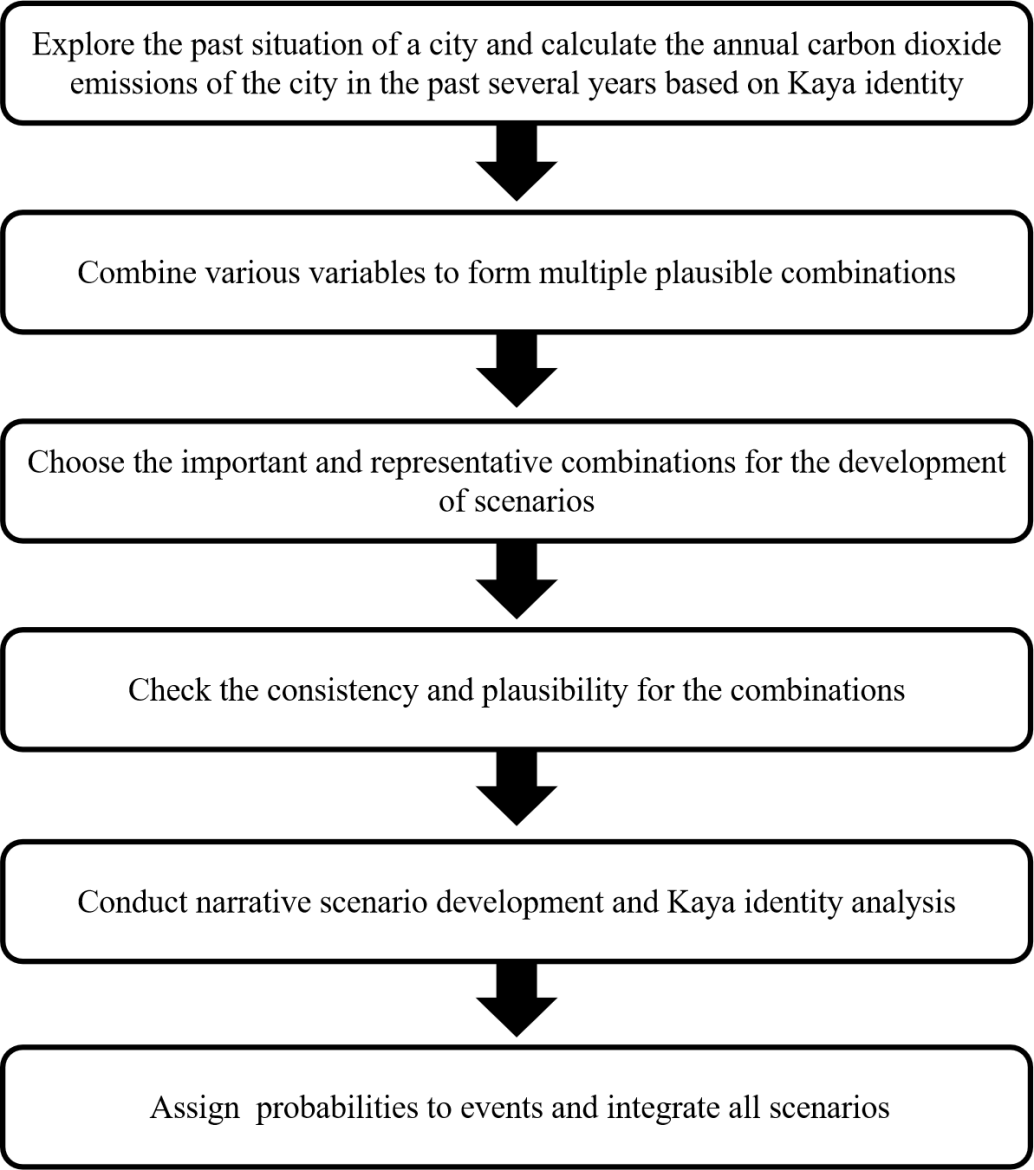
Key elements of the scenario-based Kaya identity analysis.

This study has developed a scenario-based Kaya identity methodology that capitalizes on the advantages of scenarios to navigate future uncertainty and those of Kaya identity in quantifying CO_2_ emissions. Fig 1 shows the key elements of the scenario-based Kaya identity analysis.

Japanese scholars [15,16] developed the Kaya identity (Equation 1) to link future CO_2_ emissions with population, GDP, and energy consumption changes. The Kaya identity is expressed as follows [15,16]:


$${\text{CO}}_{2}=\text{Population}\times \frac{\text{GDP}}{\text{Population}}\times \frac{\text{Energy}}{\text{GDP}}\times \frac{{CO}_{2}}{\text{Energy}}$$ (1)

where CO_2_ represents CO_2_ emissions; population represents the total domestic population; $\frac{GDP}{Population}$ represents GDP per capita; Energy represents total energy consumption; $\frac{Energy}{GDP}$ represents energy intensity; and $\frac{{\text{CO}}_{2}}{\text{Energy}}$ represents emission intensity (carbon emissions per unit of energy consumption), which is determined by the energy consumption structure.

The first element involves exploring a city’s historical context and calculating its annual CO_2_ emissions over the past several years based on the Kaya identity. Fontela and Hingel (1993) [17] considered scenarios as alternative futures resulting from a combination of trends and policies. Understanding past conditions is critical to providing a solid foundation for exploring future trends. Scenario planning combines several complex elements in a systematic, plausible, and coherent manner. The Kaya identity technique provides a scientific basis and refines the elements for developing the CO_2_ emission scenario through a quantitative analysis of CO_2_ emissions, considering important factors such as population, GDP, and energy consumption.

The second element combines the variables to form multiple plausible combinations. Zwicky [18] proposed a morphological analysis method that is being used for developing scenarios. Morphological analysis offers the advantage of systematically listing various variables and facilitating a rational combination of factors. Table 1 lists possible trends for the three factors without eliminating incompatible combinations. Going forward, brainstorming can combine different variables to form plausible futures.

**Table 1:** Morphological analysis to generate scenarios.

Variations	F1 Population change	F2 Economic development	F3 Energy consumption
Variation A	1A: Rapidly increased population	2A: Rapidly increased GDP	3A: Rapidly increased energy intensity
Variation B	1B: Slowly increased population	2B: Slowly increased GDP	3B: Slowly increased energy intensity
Variation C	1C: Population fluctuated and remained almost at the same level	2C: GDP fluctuated and remained almost at the same level	3C: Energy intensity fluctuated and remained almost at the same level
Variation D	1D: Slowly decreased population	2D: Slowly decreased GDP	3D: Slowly decreased energy intensity
Variation E	1E: Rapidly decreased population	2E: Rapidly decreased GDP	3E: Rapidly decreased energy intensity

The third element involves selecting important and representative combinations for scenario development. The combination of three factors, each with five trends, can result in a substantial number of combinations. Durance and Godet [19] argued that “importance” is a crucial condition that scenarios should address, indicating that only important scenarios must be developed. Even if it were possible to develop more than five scenarios, the cost of doing so would be high and unjustifiable. Thus, we suggest building only a small number of important and representative scenarios, based on real situations and issues that need to be addressed. Cities have different conditions; thus, developing scenarios should ensure they have the necessary expertise in urban economics, energy systems, and sociology and are familiar with the city they choose to explore.

The fourth element is plausibility and consistency check. Many incompatible and improbable combinations can be promptly produced and eliminated to avoid misleading outcomes. Morphological analysis offers a useful tool for plausibility and consistency checks [20]. A plausibility check considers if the scenarios are credible, while a consistency check examines for internal consistencies and contradictions [21].

The fifth element involves narrative scenario development and the Kaya identity analysis for each scenario. After identifying several significant and representative combinations of the variables, narrative stories can be developed based on these combinations. Relevant expertise and local knowledge of the city are key to developing such narratives. Qualitative scenarios are then linked to the quantitative Kaya identity by assigning each narrative scenario its relevant annual GDP per capita, population, energy consumption, and carbon intensity values. The combination of qualitative scenarios and quantitative analysis can leverage the advantages of qualitative scenarios by examining the interrelationships between qualitative scenario variables and the advantages of quantitative analysis for specific quantitative values for guiding better development in the future. Previous studies support the rationality underlying the link between qualitative scenarios and quantitative analysis [22–25].

The sixth element is the assignment of probabilities to events and the integration of all scenarios. However, whether probability should be used in scenario planning has been debated for long. People against the use of probability in scenario planning indicated that probability compels people to focus on the most likely future scenario rather than multiple plausible futures, destroy the story telling quality, and cause disagreement among teammates. An increasing number of researchers are using probability in scenario planning, attributing it to the advantage of avoiding neglecting important factors, exposing hidden assumptions and biases, supporting simulation, forecasting, and strategic decision making [26]. Our method includes a suggested probabilistic model as an element; however, scholars can choose to use the model based on their research objective. We encourage using data binning to assign a probability to each event and integrate all scenarios. Historical data of the studied cities and other relevant cities are the basis for determining this possibility. Morphological analysis in step 2 has divided three different factors into five variations (as shown in Table 1). Using relevant standards, numerical ranges corresponding to each variation can be defined. Calculate the frequency of occurrence in various ranges in the historical data of the relevant cities and use this as the probability of each variation appearing. Using relevant statistical methods, confidence intervals, and other information can be calculated to enhance data persuasiveness.

## 4. Scenario development

### 4.1. Setting the research questions

We are members of an eight-person Changning city-level CO_2_ emissions peak research group with extensive knowledge of CO_2_ emissions peaks and considerable familiarity with the city and its context. As an expert group, we have submitted our recommendations to the Changning People’s Government to achieve a low-carbon future. The opinions of the entire group were collected to develop the scenarios.

We developed a methodology to explore the city-level CO_2_ emission uncertainty by considering the goal of reaching a historical CO_2_ emissions peak by 2030. Four questions are presented in the case study.

1. Will the city reach its CO_2_ emissions peak by 2030?
2. How will annual CO_2_ emissions change?
3. What are the potential barriers to achieving a low-carbon future?
4. What measures can be adopted to contribute to CO_2_ emissions peak by 2030?

### 4.2. Overall context

We believe that our methodology is suitable for exploring CO_2_ emissions in every city if baseline information, such as past population, GDP, and energy intensity data, is available. These data were readily available for most Chinese cities. A county-level city, Changning in Hengyang prefecture, Hunan Province is used for our case study.

Changning is located in the southern part of Hunan Province, China, and is home to two major industries: non-ferrous metals and textile clothing production [27,28]. It is also recognized for its the reputation of being the cradle of China’s lead–zinc industry. As early as the Han Dynasty (202 BC to 220 AD) [29], residents engaged in mining activities [30].

Since the founding of the People’s Republic of China, the industrial structure of Changning has transitioned from its original focus on the smelting and chemical industries to diversification into intelligent manufacturing, electronics, apparel, and deep processing industries for agricultural products. Industrial products have shifted from being reliant on minerals and raw materials to being by the deepen processing of non-ferrous metals, with various associated value-added and derivative products developing simultaneously [31].

In recent decades, China has experienced rapid urbanization. However, research indicates that between 2010 and 2020, nearly 70% counties and county-level cities in China experienced population decline [32]. During the 13th Five-Year Plan period and the early stages of the 14th Five-Year Plan (2016–2022), Changning’s population (this study uniformly uses permanent residents as the study population rather than the household registered population) declined gradually. People moved from small towns and cities to large cities for trade opportunities, well-paid jobs, and a better quality of life [33–35]. Relevant data show that the population of Changning decreased from 834400 in 2015–786100 in 2022 [36–44]. Table 2 presents the local population for each year.

**Table 2:** Baseline population, GDP, energy and CO_2_ emissions condition in Changning.

Year	2015	2016	2017	2018	2019	2020	2021	2022
GDP (billion Chinese yuan)	26.96	29.67	33.10	33.19	33.32	35.09	40.11	43.00
Population (thousand people)	834.4	831.4	823.6	805.0	797.4	790.7	787.6	786.1
GDP per capita (Chinese yuan)	32310.6	35686.8	40189.4	41229.8	41810.9	44378.4	50926.9	54713.1
Energy intensity (ton standard coal per ten thousand yuan of GDP)	0.512	0.487	0.464	0.442	0.421	0.400	0.381	0.363
Emission intensity (ton CO_2_ per ton standard coal)	2.46	2.46	2.46	2.46	2.46	2.46	2.46	2.46
Carbon intensity (ton CO_2_ per ten thousand yuan of GDP)	1.260	1.198	1.141	1.087	1.036	0.984	0.937	0.893
Total energy consumption (thousand tons of standard coal)	1380.4	1446.2	1535.9	1466.2	1401.3	1404.9	1528.8	1560.3
Carbon dioxide emissions (thousand tons)	3395.7	3557.6	3778.4	3606.8	3447.1	3456.0	3760.8	3839.1

Although Changning’s population has declined in recent years, in the favorable macroeconomic environment of China’s rapid economic development, Changning has achieved sustained economic growth, with its GDP steadily increasing over the past decade. Thus, we collected the annual GDP and population data for Changning City from 2015 to 2022 [36–44] (Table 2) and calculated GDP per capita accordingly. We found that Changning’s GDP per capita increased from 32310.6 yuan in 2015 to 54713.1 yuan in 2022 [36–44].

To achieve the goal of a carbon peak by 2030, the Changning government intends to improve environmental capacity and energy consumption, while promoting green industrial transformation [28,45]. Changning’s total annual energy consumption between 2015 and 2022 was calculated based on GDP and energy intensity (Table 2). According to the relevant indicator target values and actual completion status in Changning City’s 14th Five-Year Plan for Economic and Social Development and the Long-Range Objectives through the Year 2035, Changning’s energy intensity was 0.512 tons of standard coal per ten thousand yuan in 2015, decreasing to 0.4 tons by 2020, indicating an approximate decline of 4.8% annually during this period [45].

According to the IPCC, the emission intensity is consistent for certain types of energy [46,47]. Energy intensity is 0.67 tons of carbon per tons of standard coal, which are the values adopted by many Chinese scholars and government authorities [48–52]. This was then converted into emission intensity, at 2.46 tons of CO_2_ per ton of standard coal. Total energy consumption per year was multiplied by the standard coal emission intensity to obtain total CO_2_ emissions per year in Changning City from 2015 to 2022, as shown in Table 2. Values in Figs/Tables are rounded to 1–3 decimal places for presentation, while actual calculations follow the methods described in this paper.

### 4.3. Changning’s CO_2_ emissions control

Over the past few years, clean energy projects have been developed in Changning City, such as the Xiaosongbai Village Photovoltaic Power Station and the Tiantangshan Wind Farm [28,53,54]. The concept of improving energy utilization efficiency and energy conservation have been effectively implemented in Changning, particularly in key sectors such as transportation, construction, and manufacturing. For example, increasing the proportion of electric vehicles [55], encouraging the development of prefabricated buildings [56], and emphasizing the efficient use of energy and pollutant emissions control for non-ferrous metal industry enterprises [57]. Industries with relatively low carbon emissions such as agriculture and services have been vigorously developed [58,59]. Afforestation activities have been effectively implemented in Changning. In 2022 alone, Changning City developed a total of 6933 hectares for forest and grass production [60]. Residents’ CO_2_ generation through various daily activities has gradually decreased.

### 4.4. Combinations of variables

**Fig 2:**
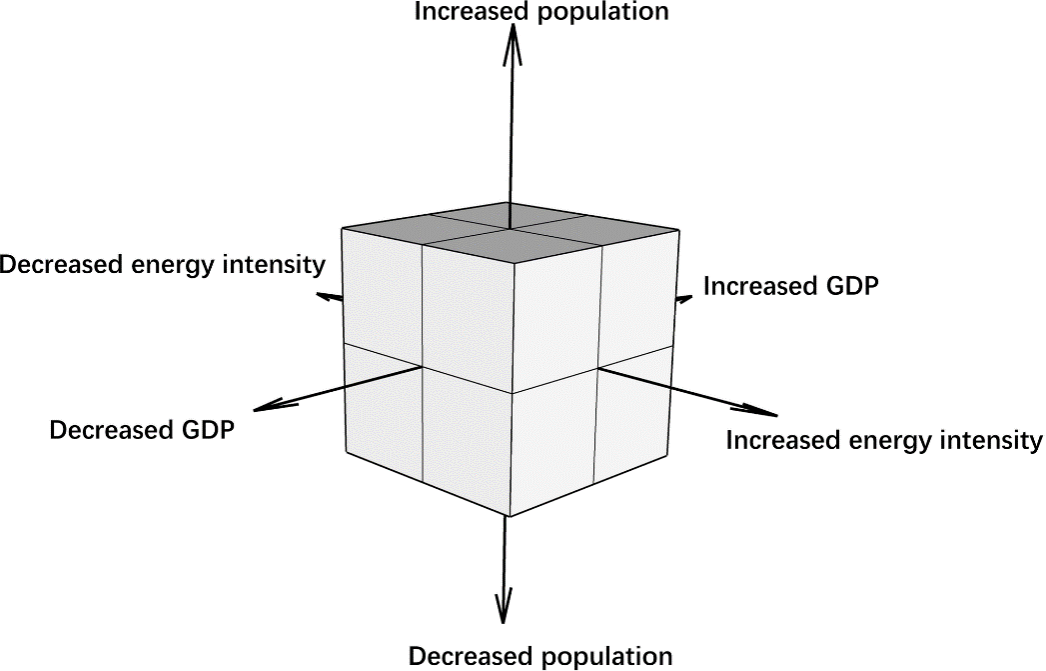
Explanation of various futures based on scenario axes.

A three-dimensional scenario axis [61–64] was developed based on the uncertainties of future population, GDP, and energy intensity, as shown in Fig 2. This axis was used to reflect on future uncertainty and facilitate the combination of various variables. From 2010 to 2020, seven counties and county-level cities of Hengyang (where Changning is located) experienced population decline [56,65]. With reference to some comparable county-level administrative units in Hunan Province, we believe that Changning’s future permanent population may decrease or remain almost as the same level, and there is a small possibility that it will increase. In 2017, China’s GDP increased by 33.5 times at constant prices compared with 1978, with an average annual growth rate of 9.5% [66]. As shown in Table 2, in the context of China’s good economic development, Changning’s GDP has grown rapidly in recent years. We believe that it is highly unlikely that a major unexpected event, such as a large-scale war, will occur in the next decade that will severely impact Changning’s GDP. Therefore, it is unlikely that Changning’s GDP will remain either at the same level or decline steadily over the next decade. Changning’s energy intensity decreased steadily during the 13th Five-Year Plan period (2016–2020) [67]. All levels of government in China are committed to achieving the goal of carbon emissions peak by 2030, and we believe that Changning’s energy intensity will not remain at the same level or could increase steadily over the next decade. Based on the above analysis, we selected only the plausible variations for the combination of variables. Table 3 shows the scenario variations, with the plausible variations marked in yellow. A combination of these variations yielded a considerable number of combinations.

**Table 3:** Selection of variations and combination of variables.

Variations	F1 Population change	F2 Economic development	F3 Energy consumption
Variation A	1A: Rapidly increased population	2A: Rapidly increased GDP	3A: Rapidly increased energy intensity
Variation B	1B: Slowly increased population	2B: Slowly increased GDP	3B: Slowly increased energy intensity
Variation C	1C: Population fluctuated and remained almost at the same level	2C: GDP fluctuated and remained almost at the same level	3C: Energy intensity fluctuated and remained almost at the same level
Variation D	1D: Slowly decreased population	2D: Slowly decreased GDP	3D: Slowly decreased energy intensity
Variation E	1E: Rapidly decreased population	2E: Rapidly decreased GDP	3E: Rapidly decreased energy intensity

### 4.5. Selecting important and representative combinations for further investigation

The business-as-usual scenario examines the consequences of continuing the current trends [68] and forms an essential point of reference for policy making [69]. In the business-as-usual scenario, we assumed that the change rates in population, GDP, and energy intensity would remain the same as the annual average change rates between 2015 and 2022. Table 4 shows the combinations of variations in the business-as-usual scenarios.

**Table 4:** Combination of variations for the development of a business-as-usual scenario.

	F1 Population change	F2 Economic development	F3 Energy consumption
Combination 1	1D: Slowly decreased population	2A: Rapidly increased GDP	3D: Slowly decreased energy intensity

In optimistic scenarios, Changning can easily achieve a CO_2_ emission peak; however, we value the importance of exploring pessimistic scenarios to determine if the Changning government can achieve its goals when encountering serious challenges. We then developed Scenario 2, which has a more challenging future for achieving the CO_2_ emissions peak in Changning. Table 5 lists the combinations of variations under the more challenging future scenarios. According to the Kaya identity, population is positively correlated with CO_2_ emissions. Thus, in a pessimistic scenario, we should assume a plausible high-population future, even if the population of many counties or county-level cities in China decreases [32]. There are some cases where the population of counties or county-level cities has increased over the past several years. For example, industrial development in Shaodong City (also a county-level city located in Hunan Province) has shown remarkable results, forming a “1+4” industrial platform pattern, including the Xiangshang Industrial Park, Lianqiao Pharmaceutical Industrial Park, Xianzhaqiao Hardware Industrial Park, and Heitianpu Printing Industrial Park. Shaodong City has experienced rapid economic growth over the past decade, stepping into the top 100 counties nationwide in terms of business environment and governance capabilities, and its resident population has increased from 896,619 in 2010–1,038,416 in 2020 [70–72]. In contrast, Changning’s population has declined steadily over the years, and we believe that it is unlikely that its population would suddenly increase by 2023. Thus, we assumed that the rate of decrease in Changning’s population would gradually decrease over the next several years because the government has adopted effective measures to reverse population decline, including rapid economic development and a large number of new jobs, which should stimulate gradual population growth first and then gradually increase.

**Table 5:** Combination of variations for the development of a more challenging future scenario.

	F1 Population change	F2 Economic development	F3 Energy consumption
Combination 2	1B: Rapidly increased population	2A: Rapidly increased GDP	3D: Slowly decreased energy intensity

Moreover, GDP is positively correlated with CO_2_ emissions. From the perspective of China’s national economic development, the annual average GDP growth rate was as high as 9.4% between 1978 and 2019 [73]. Despite the significant slowdown experienced during the outbreak of the COVID-19 pandemic, optimistic scholars have noted that China still has the potential for an annual average growth rate of approximately 8% between 2020 and 2035 [74]. Under China’s favorable economic development and rapid GDP growth conditions, some measures aimed at promoting swift economic development in Changning may be successful, including a manufacturing-led city development strategy and related strategies oriented toward chain extension, clustering promotion, and high-tech development [75]. In this context, Changning’s annual average GDP growth rate from 2023 to 2031 could be higher than its previous growth rate from 2015 to 2022. Thus, in Scenario 2, we assume a higher annual GDP growth rate.

Wen [76] noted that the reduction in energy intensity has narrowed in many provinces across China, indicating that the pressure on energy conservation and consumption reduction will gradually increase in the future, as will the cost of consumption reduction. Economic development, trade, and urban expansion can worsen environmental conditions by increasing CO_2_ emissions [77]. If the annual energy intensity in Changning decreases slowly, it will be more challenging to achieve peak CO_2_ emissions by 2030. Thus, in this scenario, we assumed a low rate of decrease in annual energy intensity.

Scenario 3 was developed based on the assumption that local governments and residents would strive to achieve their CO_2_ emission peak goal by 2030. Table 6 shows the combinations of variations under Scenario 3 (environmentalist scenario). Gazi et al. [78] highlighted that to achieve a low-carbon future, energy use should be minimized or alternated with renewable energy sources based on a comprehensive study of the relationship between macroeconomic factors and CO_2_ emissions in oil-producing countries. The superior governments of Changning attach much importance to the implementation of the Carbon Peak Action, with Changning’s government preparing the Changning Carbon Peak Action Plan and implementing active energy conservation and carbon reduction efforts [79]. We assumed that the effective implementation of energy-saving and carbon-reduction activities would lead to a steady decrease in energy intensity. For both GDP and population changes between 2023 and 2031, we assumed that no significant difference will occur and the annual average change rate from 2015 to 2022 will be maintained.

**Table 6:** Combination of variations for the development of an environmentalist scenario.

	F1 Population change	F2 Economic development	F3 Energy consumption
Combination 3	1D: Slowly decreased population	2A: Rapidly increased GDP	3E: Rapidly decreased energy intensity

Some scholars believe that as China’s economy has experienced rapid development for more than 40 years (since 1978), it has matured, and GDP growth will likely slowdown in the upcoming years [80–84]. Therefore, Scenario 4 was developed based on the assumption that Changning’s GDP growth would decelerate noticeably in the context of slowing national development. Table 7 lists the combinations of variations in the slow economic growth scenario. In this case, the rate of change of unemployment is likely to increase. We assume that when Changning’s economic development slows, many residents, especially unemployed, will move to megacities to work in pursuit of higher income levels due to unfavorable economic conditions. Therefore, we assumed a relatively rapid population decline rate in this scenario. Despite continued efforts to achieve carbon peaks, we expect the Changning government to focus on economic development because of the slowing growth. When population decline is rapid and GDP grows slowly, the government may value development and even reduce its focus on environmental protection. Therefore, this scenario assumes that the average annual decrease in energy intensity in Changning is slow.

**Table 7:** Combination of variations for the development of a slowed economy increase scenario.

	F1 Population change	F2 Economic development	F3 Energy consumption
Combination 4	1E: Rapidly decreased population	2B: Slowly increased GDP	3D: Slowly decreased energy intensity

### 4.6. Consistency and plausibility check

The key to a consistent and plausible check is to determine if the changes in population, economy, and energy intensity in Changning are reasonable. In China, most counties and county-level cities have experienced a decrease in population, whereas others have experienced an increase [32,33,70]. Future population changes in Changning could either decrease, fluctuate, remain almost unchanged, or increase, and all these scenarios are plausible. China’s GDP varies across most provinces and cities and continues to increase [85], while energy intensity continues to decrease [86–90]. Thus, it is appropriate to assume that the county area experiences population decline, even if the GDP per capita continually increases. In addition, according to Table 2, increased GDP and decreased energy intensity are past trends in Changning. It is also reasonable to assume a continuous GDP growth and decreasing energy intensity in all four scenarios. Deichmann [91] pointed out that energy intensity is negatively correlated with income growth, based on a study of 137 economies during 1990–2014. All four scenarios show an increase in GDP and a decrease in energy intensity. These four scenarios were consistent and plausible. While population size is negatively correlated with total energy consumption [92], energy intensity is largely influenced by many factors such as energy price, technological innovation, and the composition of an economic sector’s output. Moreover, an increase in the affluent population in a region may lead to an increase in energy-consuming activities, thus affecting carbon intensity [93]; however, a stable annual increase or decrease in the resident population has a limited impact on carbon intensity. The changes in population and energy intensity in these scenarios were considered reasonable. Therefore, we believe these four scenarios are consistent and plausible.

### 4.7. Narrative scenario stories

Narrative scenarios were developed based on the four combinations presented in Section 4.6. The four scenarios are business-as-usual, a more challenging future, environmentalists’ perspectives, and a slow economic increase.

#### 4.7.1. Business as usual.

Even though Changning’s economy continues to develop rapidly, some residents choose to travel to big cities such as Hengyang, Changsha, Guangzhou, and Shenzhen to pursue better working opportunities and quality of life. Moreover, even if the Changning government has tried to avoid population loss, it experienced slow population reduction. Clean energy sources such as wind, solar, and hydropower have been vigorously developed, with a continuous increase in their proportion. Energy savings and carbon reduction effects have been implemented in key sectors such as transportation, construction, and manufacturing. The proportion of electric vehicles has steadily increased, and the concept of low-carbon green travel has been effectively implemented. Moreover, the concept of green energy-saving has been implemented in the construction industry through various activities such as prefabricated buildings, and some high-polluting enterprises have been shut down. Enterprises in Changning, especially those related to non-ferrous metals and textiles, attach great importance to technological improvement and carbon emissions reduction, achieving obvious improvements in relevant carbon reduction activities. Residents of Changning actively cooperate with government initiatives to achieve the carbon peak and implement concepts such as garbage classification and low-carbon travel. Afforestation has been effective in Changning, and its energy intensity has steadily decreased.

#### 4.7.2. A more challenging future.

In the post-COVID-19 pandemic era, the Chinese economy has experienced rapid development, and the cluster effect generated by a 100-billion-yuan industry of non-ferrous metals and textiles in Changning has promoted rapid development in the area. These products are popular both domestically and internationally, with good quality and reasonable prices. Changning’s per capita GDP growth rate was ranked highest among county-level administrative units in Hengyang, gradually alleviating the impact of Changning’s shrinking population. As Changning has attracted people from nearby areas to work and engage in business, its population gradually increased after the transitional period. The Changning government has made efforts to reduce energy intensity; however, owing to the steady increase in the number of enterprises in the noted industries, the production task volume of each enterprise has gradually increased, and the carbon emission control effect related to the manufacturing industry is not noticeable. Meanwhile, Changning’s stable economic development has encouraged the development of agricultural and service industries at a relatively fast pace. People’s daily travel activities have increased, and the number of motor vehicles is growing at an accelerated pace. The Changning government is under pressure to reach its carbon peak target and avoid punitive measures if it fails. Significant and vigorous efforts may not be sufficient to effectively curb the stable growth of CO_2_ emissions. The Changning government has attempted to formulate new policies to quickly curb the growth of carbon emissions. However, the required process from introduction to implementation implies that the results cannot be achieved quickly.

#### 4.7.3. Environmentalists’ perspective.

The Changning government strongly supports the development of clean energy resources, attracting numerous enterprises to develop clean energy sources such as wind, hydro, and nuclear power. Consequently, the volume of clean energy generated has steadily increased, and the Changning government monitors CO_2_ emissions in the city annually. Strict requirements for CO_2_ emission reduction have been established for different enterprises, with a timely shutdown of those that do not meet emission standards. Enterprises, especially those in the manufacturing industry, actively use energy-saving, energy-recycling, and CO_2_ emission reduction technologies to reduce emissions. Moreover, owing to the strong government support and continuous efforts of practitioners, green agricultural products in Changning, such as tea and tea oil, have developed rapidly, promoting the city’s green and low-carbon development economy. Changning’s construction industry has shifted from rapid to efficient development, with construction projects focusing on those with high demand and contributions. The construction industry has also achieved certain energy-saving and emission-reduction goals through technological innovations, such as prefabricated construction. The concept of low-carbon travel has been well-implemented in consumer activities, with the proportion of new energy vehicles increasing rapidly, the public transportation system becoming efficient and mature, people walking and using public transportation more frequently, and a relative decrease in the use of private cars. Under the government’s active advocacy and effective leadership, public enthusiasm for participating in afforestation and forest protection is high, and the completion value of forest and grass production tasks is gradually increasing. A series of relevant activities conducted by the government has led people to focus increasingly on the importance of energy conservation and emissions reduction and to implement specific actions, such as household waste classification and low-carbon travel. Meanwhile, Changning’s economy has been developing steadily, and even thought the trend of people migrating to large cities has affected Changning’s population, the average annual rate of decline is relatively slow. On one hand, the government and people are focused on energy conservation and emissions reduction; on the other hand, various industries have the habit of starting from small to save energy and reduce emissions. As we approach 2030, the trend of a stable increase in the total annual CO_2_ emissions in Changning will be curbed.

#### 4.7.4. A slow increase in economic growth.

After more than 40 years of rapid economic development, China’s gross domestic product (GDP) growth rate has slowed significantly. The unemployment rate has gradually increased, and a slow GDP growth has intensified population outflow to large cities, resulting in a rapid decrease in the population. While striving to increase GDP growth, the government must also focus on reducing energy intensity to achieve its peak carbon goal. Thus, it promoted clean-energy power generation and established new clean-energy power stations. Although it is difficult to encourage enterprises to achieve emission reduction through energy saving, energy recycling, and CO_2_ emission reduction technologies in the context of slow economic development, the joint efforts of the Changning government and relevant enterprises have achieved certain results. Additionally, the economic downturn, the number of newly added enterprises has been relatively small, and some have shut down because of operational difficulties. Several production activities have been affected, indirectly promoting a relative reduction in CO_2_ emissions. Meanwhile, slow economic development has affected the urban construction industry, reducing the development and construction volume and construction speed. Relevant construction companies have focused on making profits while neglecting new low-carbon emission technologies such as prefabricated buildings. The development of green agricultural products—such as tea and tea oil, is also relatively slow. Under the dual pressures of slow economic development and significant population loss, the number of new motor vehicles purchased annually in Changning City is relatively small. People passively choose to use public transportation or walk during their daily travel to reduce fuel expenses. Despite significant economic pressure, the Changning government actively cultivates low-carbon concepts and encourages people to engage in low-carbon activities. Slow economic development led to reduced carbon emissions. Efforts have been made to conduct afforestation activities with relative success. Owing to the slow economic growth, a significant decline in the population, and efforts made by the government to achieve the carbon peak, the annual increase in CO_2_ emissions in Changning City is relatively slow. As 2030 approaches, the government will continue to be under pressure to achieve its peak carbon target, thereby intensifying its efforts toward energy conservation and emission reduction activities. Ultimately, the Changning government and its people worked together to effectively curb the annual increase in CO_2_ emissions in a timely manner.

### 4.8. Quantitative analysis for each narrative scenario

#### 4.8.1. GDP change.

We defined quantitative values for each scenario based on the logic detailed in the narrative scenarios and evidence-based analysis for the relevant quantitative data.

**Fig 3:**
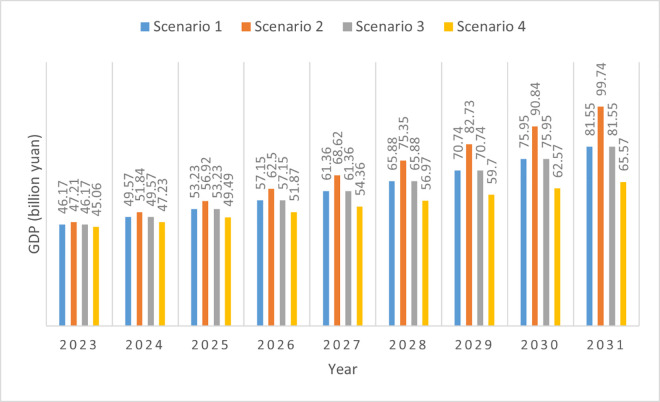
GDP change under four scenarios.

In the business-as-usual scenario, the annual average GDP increase rate remains the same as that from 2016 to 2022. We calculated the annual average GDP increase rate as 7.37% based on the rate released by The People’s Government of Changning in the annual statistical bulletin on national economic and social development, as noted in Section 4. The change in GDP in Scenario 1 (business-as-usual) is shown in Fig 3.

In Scenario 2 (a more challenging future), we assume a rapid increase in the GDP rate. The model case of Jintang has some characteristics similar to Changning; for example, the GDP for both county-level administrative units were approximately 20 billion yuan in 2012 (19.52 billion yuan in Jintang [94] and 19.89 billion yuan in Changning [44]), and both have relatively developed industries. With the backdrop of a slowed GDP growth rate in China in recent years and an annual average GDP increase rate of 7.37% in Changning over several years, we believe that it will be difficult for Changning to achieve a GDP increase rate higher than 9.8% [94] (the annual average GDP increase rate for Jintang County from 2012 to 2022). We assumed a very high annual average GDP increase rate of 9.8% for Changning over the next ten years. We believe that this scenario is beneficial for identifying potential risks in achieving CO_2_ reduction when certain factors make carbon emission goals more challenging in the context of a booming economy.

Increasing environmental protection efforts does not necessarily have a negative impact on the economy [95–97]. In contrast, increasing resource utilization efficiency and reuse rates are not only beneficial for energy conservation and carbon reduction but also for economic benefit and promoting economic development. In the environmentalists’ scenario (Scenario 3), Changning’s government considers environmental protection and achieving the carbon peak goal as crucial, while also seeking reasonable ways to vigorously develop the economy. Although stringent measures to control air pollution may lead to the shutdown of some highly polluting enterprises, Changning’s government can still find alternative methods to support the city’s economic development, such as increasing support for low-carbon technology innovation in the non-ferrous metals and textile industries, and supporting the development of planting industries such as camellia oil. This scenario also projects the average annual GDP growth rate of Changning City between 2023 and 2031 at 7.37% (similar to the annual average GDP increase rate from 2015 to 2022).

Arguments that China’s GDP growth rate will decrease further are largely attributable to the fact that China’s GDP growth rate has decreased noticeably in recent years [80–82]. If China’s overall GDP growth rate slows, it is likely that the GDP growth rates of many cities will also slow. In Scenario 4 (a slow economic growth scenario), we assume that Changning’s average annual GDP growth rate will gradually slow down. Based on the annual GDP increase rate released by the People’s Government of Changning, as well as other relevant data sources, we can see that in the past decade, Changning’s average annual GDP growth rate was 9.24% from 2013 to 2017 [98,99] and 7.02% from 2018 to 2022. The average annual GDP growth rate in Changning from 2018 to 2022 is 2.22% lower than that from 2013 to 2017. In Scenario 4 (a slow economy scenario), we assume that the average annual GDP growth rate from 2023 to 2031 would be 4.8%, which is 2.22% lower than the annual average GDP from 2018 to 2022. At an annual GDP growth rate of 4.8%, which is very low compared to the previous situation in Changning, we wanted to explore the obstacles that Changning may face in achieving its carbon peak target when economic development decelerates.

#### 4.8.2. Population changes.

**Fig 4:**
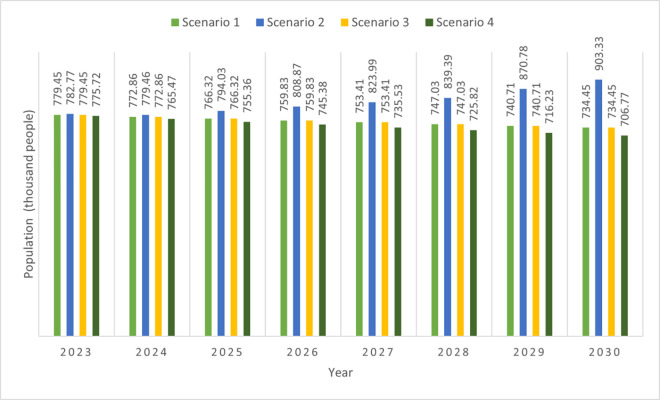
Population changes under four scenarios.

Between 2016 and 2022, Changning’s population decreased at an average annual rate of 0.846% [36,42]. In Scenario 1 (business-as-usual), we assumed that the Changning population would continue to decrease at an average annual rate of 0.846% between 2023 and 2031. As shown in Fig 4, under this scenario, the population of Changning will decrease by 58000 between 2022 and 2031.

Rapid economic development (scenario 2) was considered beneficial to alleviate population loss [100] and attract people to work and live in Changning. We believe that an average 9.8% annual increase in GDP will not lead to a sudden increase by 2023. We assumed that the annual average population decrease rate in Changning would decrease from 0.846% to 0.423% (half the annual average value between 2015 and 2022) during the first two years after 2022. We assumed that the annual average population would increase from 2025 onward. Referring to the Shaodong model case, we assumed that the annual average population increase rate in Changning would be 1.869% between 2025 and 2028 (representing half of Shandong’s population increase rate between 2010 and 2020). We also assumed that the annual average population increase rate in Changning will reach 3.739% between 2029 and 2031, which is equal to the annual average population increase rate in Shandong during the 2010s.

Stringent environmental protection does not necessarily impact trends in population change. Thus, we assumed that population changes in Changning would remain the same and decrease at an average annual rate of 0.846% between 2023 and 2031 in the environmentalist scenario (Scenario 3).

In Scenario 4 (a slow economy scenario), we assume that the population will decrease at a relatively high rate. The rationale for this is that slow economic development can lead many residents to migrate to larger cities to pursue higher salaries and better living conditions [92]. Yuanling County, another county located in Huaihua (also in Hunan Province), experienced slow GDP growth and a rapid population decline between 2010 and 2020. Referring to the average annual population decline of 1.32% in Yuanling County during the period of slow economic growth [54,101], we assumed in Scenario 4 that the average annual population decline in Changning would be 1.32% between 2023 and 2031.

#### 4.8.3. Energy intensity.

**Fig 5:**
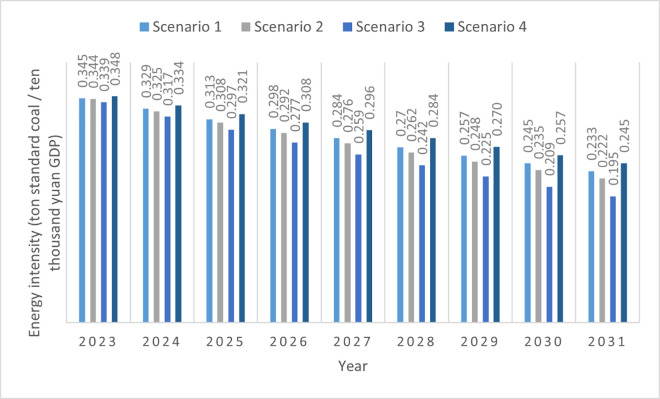
Energy intensity changes under four scenarios.

In the business-as-usual scenario, we assumed that Changning’s average annual rate of decrease in energy intensity between 2023 and 2031 at 4.8%, which is consistent with its rate during the period from 2015 to 2022. Fig 5 shows the annual change in energy intensity in this scenario, which indicates that the energy intensity will decrease from 0.345 tons of standard coal per 10000 yuan in 2023 to 0.233 tons in 2031.

In the IPCC guidelines, the carbon emission factor method provides a fixed value for emission intensity. The reduction in CO_2_ emissions during the transition from fossil energy to renewable energy sources is reflected in the increased use of clean energy, which is conducive to improving energy efficiency and thus reducing energy and carbon intensities [12,102]. As mentioned in the narrative scenarios, the limited success in increasing the use of clean energy and improving energy efficiency in Scenario 2 did not lead to a rapid decrease in energy intensity during the period of rapid economic development. We refer to cases in which GDP growth is fast but has limited success in improving energy efficiency. In the more challenging future (Scenario 2), we refer to Ningyuan, a county with a high GDP growth rate during the 13^th^ five-year plan period (2016–2020), during which its annual average rate of decrease in energy intensity was 5.3% [103]. Thus, we assumed a similar 5.3% rate of change between 2023 and 2031 because of the effectiveness of relevant strategies to reduce carbon emissions during the economic boom period.

In the environmentalists’ scenario (Scenario 3), the narrative specifies that the government will make significant efforts to control CO_2_ emissions through comprehensive policy interventions in various fields, such as traffic, industry, construction, forestry, and agriculture. Changning citizens will also endeavor to decrease their carbon footprint from work to daily life. In this case, energy consumption could soon be reduced. We assumed a high rate of decrease in energy intensity in this scenario. Shimen is a good model of CO_2_ emissions. It is a county-level administrative unit located in Changde (Hunan Province) and has vigorously implemented a strategy of building an ecological county and strengthening itself through industry, regarding industrial transformation as a revolutionary measure for building an ecological county. Shimen County highlights the establishment of green and circular transformation brands, building a transformation platform around the industrial park to improve quality, and gives full play to the driving effects of flagship enterprises such as Datang, Conch, and Huadian to promote green and circular development. Renewable energy has become the main source of development in Shimen’s recycling economy [104]. During the 2015–2020 period, Shimen achieved an annual average energy intensity reduction rate of 6.5% [105]. The environmentalist scenario assumes that Changning’s government and people will make significant efforts to develop a low-carbon economy in the future and achieve a 6.5% annual average decrease in energy intensity from 2023 to 2029. To achieve the peak carbon emissions goal between 2029 and 2031, the Changning government worked hard to reduce CO_2_ emissions, and the annual average rate of decrease in energy intensity reached 7%.

In the slow economic development scenario, we assume a relatively low rate of decrease in energy intensity because it correlated negatively with income growth [91]. Even if the Changning government experiences difficulties in developing a low-carbon economy, such as lack of funding to support technological innovation to achieve renewable energy transformation, encountering difficulties in improving energy use efficiency, and rapid annual energy use increase in different sectors, it will make significant effort after achieving CO_2_ emission peak as a nationwide goal and should be valued. During periods of slow economic development, people focus on economic development and may focus less on environmental protection. We refer to Huarong County (Yueyang, Hunan Province) as the model case. It had a relatively low GDP growth rate of 35.7% between 2015 and 2020 (annual average growth of 5.2%) and a relatively low rate of 4% decrease in energy intensity [106]. Thus, we assumed that the reduction rate of energy intensity in Changning would be 4% from 2023 to 2028, which is lower than the average value between 2015 and 2022. Changning’s government and people value the importance of achieving the carbon emission peak in 2030 and will strive to achieve this goal between 2029 and 2031, where the reduction rate of energy intensity will be 4.8% between 2029 and 2031 (reaching Changning’s same annual average rate of decrease between 2016 and 2020).

#### 4.8.4. Carbon intensity.

**Fig 6:**
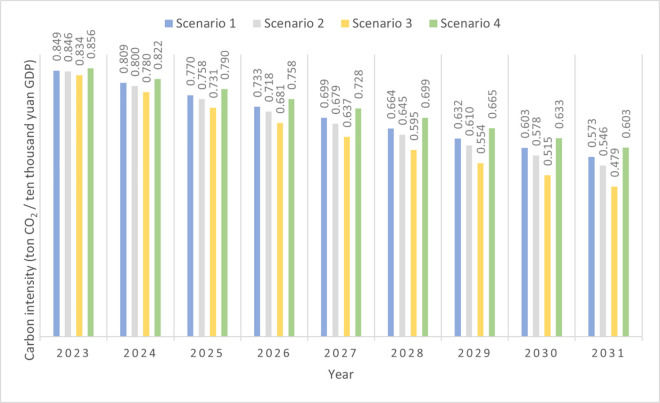
Carbon intensity changes under four scenarios.

As shown in Fig 6, carbon intensity decreased annually across the four scenarios. This trend aligns with China’s national efforts to improve energy efficiency and reduce carbon emissions and the gradual decline in China’s overall carbon intensity.

In Scenario 1 (business-as-usual), the carbon intensity reduction rate ranked third among the four scenarios, decreasing from 0.849 tons of CO₂ per ten thousand yuan of GDP in 2023 to 0.573 tons in 2031. This demonstrates the need for additional efforts to enhance energy efficiency, reduce emissions, and ensure to realize the peak carbon target under the current policy framework.

In Scenario 2 (a more challenging future), despite rapid GDP growth and the positive correlation between GDP and CO₂ emissions, as described by the Kaya identity, extensive implementation of China’s 2030 carbon peak policies will exert a profound impact on various cities. During periods of rapid economic growth, the Changning Municipal Government is expected to intensify policy measures and increase financial support to achieve its carbon peak targets. Consequently, in this scenario, carbon intensity decreases significantly from 0.846 tons of CO₂ per ten thousand yuan of GDP in 2023 to 0.546 tons in 2031.

In Scenario 3 (environmentalist scenario), Changning consistently achieved the lowest carbon intensity among all scenarios. Under this scenario, the city’s carbon intensity will decrease from 0.834 tons of CO₂ per ten thousand yuan of GDP in 2023 to 0.479 tons in 2031. This underscores the critical importance of comprehensive efforts to enhance energy efficiency and reduce emissions to achieve the city’s carbon peak target.

Conversely, in Scenario 4 (slow economic development), carbon intensity remained high with a slow reduction rate. Between 2023 and 2031, carbon intensity will likely drop from 0.856 tons of CO₂ per ten thousand yuan of GDP to 0.603 tons. This indicates that in this scenario, a slower increase in annual CO₂ emissions is largely attributable to lower GDP growth. Hence, even when GDP growth is slow, it is essential to prioritize energy efficiency and carbon emissions reduction efforts. This is crucial to foster low-carbon development and mitigate global climate change.

#### 4.8.5. GDP per capita.

**Fig 7:**
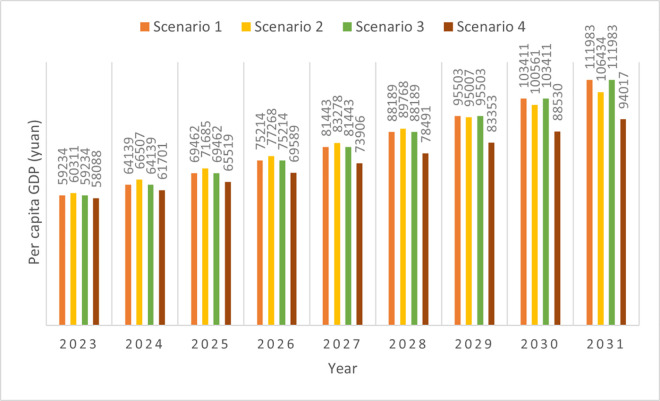
GDP per capita changes under four scenarios.

Our case study focused largely on exploring the possible changes in Changning’s future GDP and population than on exploring the changes in both GDP per capita and population. However, our developed methodology can also focus on a city’s GDP per capita and construct scenarios based on it, and the resident population size and energy intensity, to explore multiple possible futures. Fig 7 shows the changes in the GDP per capita under the four scenarios.

In Scenario 4 (slow economic development), the annual GDP per capita was the lowest among the four scenarios. Owing to the rapid GDP growth between 2023 and 2028 in Scenario 2, it has the highest GDP per capita value among the four scenarios. In Scenario 2, it was assumed that population decline in Changning would gradually slow and then begin to increase. Owing to a noticeable increase in Changning’s population between 2029 and 2031 in Scenario 2, its GDP per capita growth rate value is lower than the value from 2023 to 2028. This supports the argument that rapid population growth can have a negative influence on GDP per capita growth [107–109].

#### 4.8.6. CO_2_ emissions.

**Fig 8:**
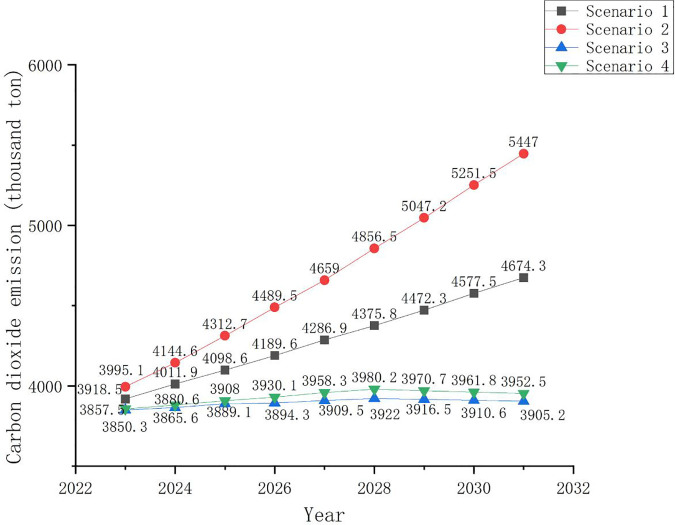
CO_2_ emissions under four scenarios.

Fig 8 shows the CO_2_ emission changes under the four scenarios. According to Scenario 1, if the trends in Changning’s population, GDP, and energy intensity remain consistent with those of the previous seven years, Changning City will not reach its carbon peak before 2030. Changning’s CO_2_ emissions will steadily increase from 3918.5 kilotons in 2023 to 4674.3 kilotons in 2031. Thus, the Changning government and people must increase their efforts in energy conservation, emissions reduction, and low-carbon economic development.

According to Scenario 2, Changning’s annual CO_2_ emissions will increase from 3995.1 kilotons in 2023–5447 kilotons in 2031. This indicates a close relationship between GDP growth, population change, and CO_2_ emissions. If only the reduction in energy intensity through various means is emphasized, while ignoring the impact of GDP and population changes on carbon emissions, it is likely that a city will face a situation wherein CO_2_ emissions increase rapidly.

Under the environmental scenario (Scenario 3), CO_2_ emissions slowly increase from 2023 to 2028. In 2028, CO_2_ emissions will only be 71.7 kilotons higher than those in 2023. As the target year of the carbon peak approaches, people will further increase their efforts toward energy conservation and emissions reduction, effectively achieving the CO_2_ emission peak in 2028. Therefore, under the premise of combining economic development with a strong emphasis on low-carbon development, Changning could achieve its peak carbon emissions by 2030.

In Scenario 4, owing to the slow GDP growth rate, even if Changning’s energy intensity decreases relatively slowly, it could maintain a slow increase in CO_2_ emissions between 2023 and 2028. Thus, CO_2_ emissions in 2028 will only be 122.7 kilotons higher than those in 2023. In Scenario 4, the annual decline in energy intensity from 2023 to 2031 will not exceed 4.8% of the annual decline in energy intensity from 2016 to 2022. However, in this scenario, changes achieve a carbon emissions peak before 2030. The carbon peak achieved in this manner may lead to a resurgence in CO_2_ emissions in the future owing to rapid economic growth and insufficient environmental protection efforts.

### 4.9. Probability assignment

Our scenario development did not end with a quantitative analysis. This section attempts to assign a probability to each event and integrate all scenarios.

We referred to the annual historical data (GDP and population) of seven counties or county-level cities (Changning City, Leiyang City, Hengyang County, Hengnan County, Hengshan County, Hengdong County, Qidong County) belonging to Hengyang City between 2015 and 2022 to explore the probability of GDP and population change in each scenario. Owing to similarities in policy environments, demographic trends, and even industrial structures, the six county-level units in Hengyang exhibited high socioeconomic similarity to Changning, supporting the pooled data analysis for projections.

We selected 20 county-level administrative units (including county-level cities and counties) located in Hunan Province based on characteristics such as economic structure, population changes, industrial transformation processes, resource endowment conditions, urbanization level, and policy support. We collected the average annual energy intensity reduction data for these administrative units during the 13th Five-Year Plan (2016–2020) period, which served as the basis for judging the frequency of variations in energy intensity in Changning’s scenario planning. The city names and specific data mentioned above are provided in the supporting information.

**Fig 9:**
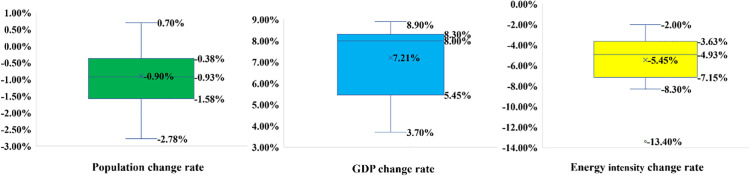
Box plots based on referenceable historical data.

Box plots (Fig 9) are based on historical GDP, population, and energy intensity data. These formed the basis for calculating the probabilities and confidence intervals for each variation in each factor. Due to many county-level administrative units directly using national census population data in 2020 in their official report, the annual population estimation methods of the national census and county-level administrative units may be different, which could lead to some discrepancies in the calculation of permanent population decline based on official data this year, such as a high decrease rate of 13.06%. Therefore, the probability estimation of permanent population decline was based on data between 2015 and 2019 and between 2021 and 2022 (seven years). It can be seen from Fig 9 and the supporting information that the historical population change rate, which can be used as a reference, includes both population increases and decreases. Based on the actual situation, according to the box plot, a decrease below the median value (−0.93%) is defined as a rapid decrease between −0.93% and −0.38% (the upper quantile) is defined as a slow decrease. A small portion of the historical data shows that some relevant county-level administrative units have experienced an annual population increase. The threshold was determined based on the relevant threshold of the population decrease. A change rate between −0.38% and 0.38% is defined as a fluctuation and remains at the same level, a growth rate between 0.38% and 0.93% is defined as slow growth, and a growth rate above 0.93% is defined as rapid growth. Table 8 shows the probabilities, averages, and confidence intervals for each variation in population change.

**Table 8:** Probability, average value, and confidence interval of each variation.

Factors	Variations	Values	Probability	Average value and confidence interval (95%)
Population	1A: Rapidly increased population	>0.93%	2%	None (a single data point)
	1B: Slowly increased population	≤0.93% and >0.38%	8.2%	0.605% ± 0.175%
	1C: Population fluctuated and remained mostly at the same level	≤0.38% and ≥−0.38%	14.3%	0.0286% ± 0.259%
	1D: Slowly decreased population	<−0.38% and ≥−0.93%	26.5%	−0.628% ± 0.097%
	1E: Rapid decreased population	<−0.93%	49.0%	−1.671% ± 0.256%
GDP	2A: Rapidly increased GDP	>7.21%	71%	7.21% ± 0.48%
	2B: Slowly increased GDP	≤7.21%	29%	4.70% ± 0.38%
Energy intensity	3E: Rapidly decreased energy intensity	≤−5.45%	35%	−8.26% ± 2.18%
	3D: Slowly decreased energy intensity	>−5.45%	65%	−3.94% ± 0.66%

The average value of referred historical GDP change rates is 7.21% (Fig 9) and is regarded as the division line between high and low GDP growth rates. Among the 49 historical annual GDP growth rate data available for reference (seven counties, each county’s GDP growth rate for seven years (2016–2022); specific values can be found in the supporting information), 29% were below the average of 7.21%, and the remaining 71% were above the average. Using this as a standard, the probabilities of low and high GDP growth rates were determined to be 29% and 71%, respectively, and the mean and confidence intervals of the high and low growth rates were further calculated, as shown in the Table below.

As shown in Fig 9, the average value of the 20 energy intensity reduction rates available for reference is −5.45%. We define data with a decrease rate lower than this value as rapid decrease in energy intensity and above this mean as slow decrease in energy intensity. Nearly 35% of the 20 data points were in the high energy intensity reduction range, whereas 65% were in the low energy reduction range. We take this as the probability of low and high energy intensity and further calculate the mean and confidence interval of the data in the two intervals, as shown Table 8.

By sequentially determining the probabilities of each variation in each factor of the four scenarios from Table 8 and multiplying the probabilities of each variation in each scenario, the probability of each scenario appearing for reference can be obtained (Table 9). By comparison, it can be concluded that the scenario with the highest probability is business as usual. This also inspires government departments not to be satisfied with the current rapid decrease in energy intensity but to continuously increase environmental protection efforts. According to the calculations presented in Section 4.8, this scenario could not achieve a carbon peak before 2030.

**Table 9:** Probabilities of scenarios.

Scenario name	Business-as-usual	A more challenging future	Environmentalists’ perspective	A slowed economic increase
Probabilities	12.23%	0.92%	6.59%	9.24%

The probability of a more challenging future was the lowest (0.92%). This can be explained by the carbon peak being a nationwide policy in China and county-level administrative units attaching significant importance to achieving this goal under various pressures. When the economy develops rapidly, government departments increase environmental protection efforts and invest in environmental protection funds to achieve their carbon peaking goals. Although the probability of this scenario is low, it is significant. This scenario has the highest carbon dioxide emissions. High-risk scenarios with a low probability of occurrence should also be considered.

## 5. Sensitivity analysis

**Fig 10:**
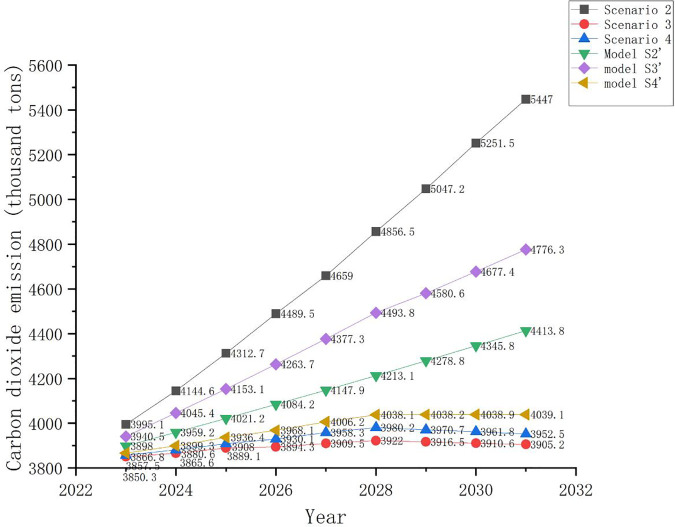
A comparison of scenarios 2, 3, and 4 and three sensitivity analysis models.

Sensitivity analysis was conducted to understand how variations in population, GDP, and energy intensity may affect CO_2_ emissions. We built Models S2’, S3’, and S4’ based on scenarios 2, 3, and 4, respectively, to explore the influence of these parameters on CO_2_ emissions. Fig 10 shows the four scenarios and the three sensitivity analysis models.

Owing to the inverse relationship between energy intensity and income [91], rapid GDP growth may lead to a faster decrease in energy intensity than that proposed in scenario 2. Model S2’ assumes the same trend of GDP and population changes as in Scenario 2 but with an annual reduction of 7.5% in energy intensity. The GDP of Hanshou County, Changde City, Hunan Province grew rapidly between 2016 and 2020, with an average annual growth rate of 7.6% and an average annual decrease of 7.5% in energy intensity [110]. Therefore, under the premise of rapid economic development, we believe that change may achieve a significant reduction in energy consumption. Comparing Scenarios 2 and S2’ in Fig 10, although different reductions in energy intensity can lead to significantly different CO_2_ emission levels, owing to Changning’s rapid increase in GDP in Scenario 2, the decrease in energy intensity does not exceed a specific critical value and does not change the current growth in annual CO_2_ emissions.

As there is an inverse relationship between energy intensity and income [91], we believe that a higher GDP increase rate and an annual average decrease rate in energy intensity of 6.5% between 2023 and 2028 and 7% between 2029 and 2031 are still reasonable. We assume that the GDP increase rate in Model S3’ will be 9.8% between 2023 and 2031 (an increase rate equal to that of the model case of Jintang County from 2012 to 2022) [94], and the annual average population decrease rate and annual average decrease rate in energy intensity will still be 0.846% between 2023 and 2031, 6.5% between 2023 and 2028, and 7% between 2029 and 2031, respectively. Fig 10 shows that Changning will not be able achieve the CO_2_ emission peak before 2030 in Model S3’. This shows that GDP has a considerable influence on CO_2_ emissions. For a certain annual average increase rate in GDP, only the energy intensity decrease rate reaches a correspondingly high level, and the city’s CO_2_ emissions will decrease.

To better explore the impact of a city’s overall economic development on carbon emissions, we used GDP rather than GDP per capita or population to analyze carbon emissions in the previous sections. Model S4 explores the impact of population changes on CO_2_ emissions from the perspectives of GDP per capita, population, and energy intensity. Model S4’ was developed based on Scenario 4 but assuming a slower decrease rate in population, the same annual increase rate in GDP per capita, and a reduction rate in energy intensity as in Scenario 4. We used an average value of 0.846% (the average annual population decrease rate in the business-as-usual scenario) and 1.32% (from the slowed economic development scenario) as the annual average population decrease rate in Model S4’ for this slowed economic development with a moderate population decrease model. The rate of decrease was calculated to be 1.083%. We calculated the CO_2_ emissions in Changning based on the annual GDP per capita increase rate, population, and energy intensity. In Model S4’, we found that Changning will not achieve its CO_2_ emission peak before 2030. This indicates that population influences CO_2_ emissions significantly.

## 6. Discussion and policy implications

### 6.1. Main features of the method

A main feature of the scenario-based Kaya identity analysis is that it directly considers the three important variables in the Kaya identity (GDP or GDP per capita, population size, and energy intensity) as the three uncertainty factors in scenario planning, closely linking scenario planning and the Kaya identity. The advantage of the scenario-based Kaya identity approach is that it can help city managers and other relevant stakeholders consider setting targets for reducing energy intensity under different GDP and population change scenarios. Based on this analysis, a series of targeted policies can be introduced to promote the realization of the carbon peak in Changning.

### 6.2. Policy suggestions

#### 6.2.1. Policy suggestions based on the business-as-usual scenario.

We believe that if changes in Changning’s GDP and population between 2023 and 2031 maintain the average annual change rate from 2016 to 2022, the government will set the target of reducing energy intensity during the first stage between 2023 and 2028 at 6% to achieve the carbon peak before 2030. This will significantly reduce the annual CO_2_ emissions in Changning, and for each year during the 2023–2028 period, only 30–40 tons more CO_2_ emissions will be produced than in previous years. In the second stage (2029–2031), the target for reducing energy intensity was set at 7%. Thus, changes could achieve a carbon peak before 2030.

Stringent peak carbon policies or strategies must be developed to gain greater control over annual CO_2_ emissions. Changning should vigorously promote the Shanmi Chong pumped-storage power stations, support the expansion and continuation of wind power projects such as Baisha and Yangquan, and develop rooftop solar power projects at government, school, and hospital buildings. The Changning government should intensify efforts to close highly polluting industrial enterprises and encourage relevant enterprises to use clean energy, such as transitioning from coal to gas and electricity. Resource utilization efficiency can also be increased by promoting industrial solid waste recycling, fully utilizing industrial waste heat, and accelerating the development of green and low-carbon industries, such as agricultural product processing, textiles, and electronics. Moreover, energy- and water-saving development in urban and rural construction, renovation of old buildings, and increasing support for energy-saving, material-saving, and carbon-reducing prefabricated buildings, including the prioritization of prefabricated building development through funding or project approval support, will be particularly helpful in the construction sector. More funds should be allocated to promote carbon-reduction technologies. The Changning government should actively promote the construction of low-carbon smart infrastructure, such as charging stations, and encourage citizens to purchase clean-energy vehicles and use low-carbon transportation. Increased afforestation efforts will enhance the carbon sequestration capacity in forestry, agriculture, wetlands, and other areas. Moreover, strengthening public low-carbon publicity and education initiatives, guiding enterprises to fulfil their social responsibilities, actively participating in the green trading market, and implementing the policy orientation of people who pollute should be responsible for taking action to reduce CO_2_ emissions can increase the likelihood of meeting the carbon peak goal.

#### 6.2.2. Policy suggestions based on the more challenging future scenario.

Assume that Changning’s annual GDP growth rate is 9.8% between 2023 and 2031. In this case, the energy intensity decrease rate must be set at 8% annually during the 2023–2028 period, and the annual CO_2_ emissions will increase slowly compared with the previous year. For each year from 2023 to 2028, only 35–45 tons more CO_2_ emissions will be produced compared to previous years. In the second stage (2029–2031), the target for reducing energy intensity should be set at 9%. Thus, this change could achieve its carbon peak before 2030.

With the rapid pace of economic development, the Changning government needs to implement stringent measures to save energy and reduce carbon emissions to achieve its CO_2_ emission peak. If the government does not attach importance to low-carbon economic development, it is likely that the CO_2_ emission peak will not be reached because of the government’s inability to achieve a corresponding rapid reduction in energy intensity. In the context of rapid economic development, Changning’s government can fully utilize the advantages of good economic conditions to increase government regulations by creating new policies and strategies and setting higher goals for enterprises and citizens. They could also leverage the advantages of rapid economic development to vigorously develop renewable energy by accelerating the construction of the Shanmi Chong hydropower station, attracting capital to build more wind power stations, and taking specific measures to encourage companies and households to use photovoltaic power generation. Moreover, governments can provide greater support for green and low-carbon technological innovation by strengthening innovation capacity building and talent acquisition. Higher goals for low-carbon transformation can be set in various industries, further increasing the support for low-carbon transformation, such as encouraging enterprises to increase R&D efforts in carbon reduction and setting higher requirements for industrial enterprises to transform their energy consumption methods. Changning People’s government can leverage the advantages of economic prosperity to attract more low-carbon industries, such as agricultural product processing and electronic equipment manufacturing enterprises. These industries are not only beneficial for Changning’s economic development but also for its low-carbon transformation. The Changning government can gradually increase its green buildings and prefabricated building projects annually as the GDP grows rapidly, and spend more money to support the construction of low-carbon and smart infrastructure. Citizens should be encouraged to purchase electric vehicles and promote low-carbon travel in addition to investing in promoting green and low-carbon concepts. Rapid economic development also means that the Changning government and related enterprises can allocate more funds toward afforestation activities.

#### 6.2.3. Policy suggestions based on environmentalists’ scenario.

In the environmentalists’ scenario, we found that when energy intensity remained unchanged (consistent with Scenario 3), and the annual GDP growth rate increased from 7.37% to 7.5%, Changning can still reach its carbon peak before 2030. However, when the annual GDP growth rate increases to 7.6% or higher, Changning cannot achieve the CO_2_ emission peak.

This environmentalists’ perspective scenario indicates that environmental awareness of the government and people is crucial for achieving the carbon peak in Changning. Scenario 3 shows that the Changning government can enhance the responsibility of its departments, corporations, social organizations, and improve the general public’s environmental awareness to achieve the carbon peak by formulating and implementing a series of policies and strategies. These can unleash supervisory agencies to enable the government and people to work together to achieve the peak carbon goal. After developing good policies to maximize the relevant government departments’ sense of responsibility and people’s environmental awareness, the Changning government can further develop appropriate policies and strategies to promote economic development.

Our policy and strategy recommendations are as follows. Higher-level governments of Changning (the Central People’s Government of the People’s Republic of China, the People’s Government of Hunan Province, and the Hengyang Government) have formulated several policies and strategies to enhance the responsibility of government departments, corporations, and social organizations, and the public’s environmental awareness. The Changning government should formulate corresponding policies and strategies based on its specific local context, while enforcing the relevant policies and systems proposed by higher-level governments. For example, the Hunan Provincial Government has set the goal that prefabricated building areas should account for more than 40% of the newly built construction area by 2025 for low-carbon development and mandates that government-invested buildings with a construction area of more than 3,000 square meters and other public buildings with a construction area of more than 20,000 square meters to adopt prefabricated construction or other green construction methods [111]. In addition, a distinctive goal should be formulated based on the characteristics of Changning’s numerous metal smelting and textile factories: by 2025, at least 50% of the metal smelting and textile factories should be housed in prefabricated buildings. This policy is conducive to encouraging Changning’s well-developed metal smelting and textile factories to fully utilize their vital financial resources to increase innovation and investment in factory construction. Good practices for low-carbon development in other cities can also guide changes in policy formulation regarding the carbon peak goal. For example, as a resource-exhausted city [112,113], Changning can fully draw on the relevant low-carbon development efforts of Baiyin City, which is another resource-exhausted city in China. As such, they should promote the construction of comprehensive utilization and centralized disposal facilities for bulk hazardous waste (aluminum ash, chromium slag, garbage incineration fly ash, etc.), mercury-containing waste (mercury-containing fluorescent lamps), and lead-containing waste, effectively addressing the limitations in utilization and disposal [114–116].

#### 6.2.4. Policy suggestions based on a slowed economic development scenario.

In the context of China’s slow economic development and its significant impact on the economy and severe population loss, we recommend setting a target to control the annual population decline to 0.846%, that is, not exceeding the average yearly decrease from 2016 to 2022. Meanwhile, the government should strive to increase the annual GDP growth rate to approximately 6% and maintain a 5% decrease in energy intensity between 2023 and 2028 and 6% between 2029 and 2031. During an economic downturn, the Changning government should set reasonable goals, strive to develop its economy, and reduce CO_2_ emissions.

From Scenario 4, we can see that in the upcoming years, even if Changning’s average annual GDP growth rate slows. The government prioritize energy conservation and carbon emission reduction. Otherwise, changes may lead to an annual increase in carbon emissions in the future due to rapid economic development. If the Changning economy develops slowly and population loss becomes more severe, the government should focus on the path of low-carbon economic development. In cases of slow economic development, many enterprises face the risk of reduced production or bankruptcy. The Changning government should strive to introduce enterprises with lower carbon emissions and provide policy support for relevant enterprises. The government can promote the development of typical primary industries such as tea oil, strawberries, and ginger by formulating relevant policies. Policy intervention is effective in influencing the agricultural commodities market [117]. The government can actively help with agricultural product sales. For Changning’s two pillar industries of metal smelting and garments, the government can guide and encourage them to increase innovation efforts and adopt the path of low investment, especially low energy consumption and high-efficiency development, based on their own situation through a series of policy support. This will help reduce investment and increase production. Further support for the sale of clean energy vehicles can be achieved through preferential policies such as tax reductions, loan incentives, and financial subsidies for the development of clean energy vehicle companies in Changning. For renewable energy infrastructure, the government can formulate relatively loose policies to attract private capital investment. The government should consider investing appropriate public funds to stimulate the development of the construction industry, particularly low-carbon green and prefabricated buildings. They can also promote low-carbon development in construction through credit, corporate bonds, private equity funds, and green-building performance liability insurance. The government can conduct a series of activities to promote low-carbon development through relatively low-cost but high-return methods, including encouraging local primary and secondary schools to offer low-carbon education classes, local radio and television programs, and organizing low-carbon publicity and theme activities on special holidays, such as Arbor Day. It can also encourage the establishment of low-carbon environmental volunteer organizations, guide them to conduct appropriate activities, make more people aware of the importance of reducing carbon emissions, and encourage environmentalists to work together in a common organization.

### 6.3. External validity

Our case study shows that the developed methodology can help local governments to consider future CO_2_ emissions and develop strategies. This methodology is particularly useful for achieving energy intensity reduction goals under various GDP and population change scenarios.

Changning is a typical resource-exhausted city in China with a strong industrial foundation that gives it a high GDP growth rate and low population change rate. During the exploration period of consolidating the deep processing industry of products and transforming them into agriculture and service industries, Changning faced both opportunities to promote the rapid development of a low-carbon economy through rapid changes and challenges from weak foundations in the primary and tertiary industries. This will undoubtedly have a significant impact on Changning’s future low-carbon development path. As in the environmentalists’ perspective scenario, finding a development path that can maintain stable and rapid economic growth while being low-carbon and environmentally friendly will be the focus and difficulty for the Changing government and people during this special transition period. All resource-exhausted cities face similar opportunities and challenges in the process of low-carbon development. Therefore, like Changning, they must transcend the status quo during the special period of industrial transformation and seek economic models based on low energy consumption, low emissions, and low pollution, while pursuing stable and rapid economic growth by implementing appropriate and efficient changes. The government’s macroeconomic regulation is crucial during this transitional period, increasing funding and support for scientific research and development, and implementing stringent regulatory policies for promoting industrial transformation and upgrading high-energy-consuming and high-emission industries. The role of non-governmental organizations, such as environmental protection associations, should be leveraged, encourage the development of associations to allow other environmentalists to join this collective and give relevant associations more supervisory and decision-making authority to encourage ordinary people to play an important role in achieving low-carbon development goals. In addition, other local governments should value the importance of high-risk events even if the probability of a sudden and rapid increase in the economy is low. This will make it much more difficult to achieve the CO_2_ peak. Environmental awareness and actions by the government and public are crucial in the carbon peaking process. It is not wise to achieve carbon peaking goals by sacrificing economic development. Developing a low-carbon economy will enable to achieve a win-win situation. It is important to realize that the carbon peak is a gradual process and rushing it may increase CO_2_ emissions instead of decreasing.

We encourage placing greater emphasis on developing deep processing of metal products, clothing production, and the development of agricultural products such as tea oil, strawberries, and ginger that can adapt to local climatic conditions. However, this industrial transformation approach may not apply to other resource-exhausted cities. The effectiveness of developing the above-mentioned industries for economic development and reducing carbon dioxide emissions needs to be evaluated over time. At the same time, although measures such as increasing clean energy generation, supporting the development of prefabricated buildings, and promoting the use of new energy vehicles are suitable across all cities to reduce carbon emissions, the appropriate behaviors to achieve these goals may vary for different cities. For example, we believe that Changning can vigorously develop hydropower, whereas some cities may be more suitable for developing wind power. Additionally, the most suitable policies for supporting prefabricated building development may differ across cities.

## 7. Conclusion

We developed a scenario-based Kaya identity analysis methodology to explore CO_2_ emissions. It relies heavily on GDP, population, and energy intensity. By analyzing the interrelationships among the key factors, we can develop multiple future scenarios to inform targeted policies and strategies for achieving urban CO_2_ emission reduction goals.

Based on Scenario 1, Changning should develop stringent policies in six major fields–energy, secondary industry, urban-rural building construction, transportation, agriculture, rural economy and four major industries including steel, non-ferrous metals, petrochemical and chemical industries, and building materials. Based on the analysis of Scenario 2, we found that if Changning’s economy develops rapidly over the next few years, the government must leverage this scenario, increase government regulations based on existing policies, formulate stricter policies, increase government funding support, reduce high-polluting enterprises, and introduce environmentally friendly enterprises. It should also fully leverage the subjective initiatives of public institutions, enterprises, social organizations, and the public to reduce carbon emissions and rapidly decrease Changning’s energy intensity. In Scenario 3 analysis, we found that a number of factors are crucial for reducing CO2 emissions: increasing the responsibility of relevant government departments to achieve carbon peak goals; fully leveraging the role of public institutions, enterprises, and social organizations in energy conservation and carbon reduction; and increasing the environmental awareness among Changning’s citizens to reduce carbon emissions. Based on Scenario 4, we found that in the upcoming years, if Changning’s economy grows at a conspicuously slow pace, the government should formulate relevant policies to strongly support the development of a low-carbon economy and steadily reduce citizens’ carbon footprint.

Our methodology can be used to support researchers, government consulting teams, and decision-makers in considering future CO2 emissions and facilitating strategic decision-making.

## Supporting information

S1 File Energy intensity change rate of Shimen. (DOCX)

S1 Table The annual GDP change rate of seven county level administrative units of Hengyang from 2016 to 2022. (DOCX)

S2 Table The annual population of seven county level administrative units of Hengyang from 2014 to 2022. (DOCX)

S3 Table The annual population change rate of seven county level administrative units of Hengyang from 2015 to 2022. (DOCX)

S4 Table The annual average energy intensity change rate of twenty county level administrative units in Hunan province from 2015 to 2020. (DOCX)
