# Supplementary material for: Scenario-based Kaya identity analysis for city-level carbon dioxide emissions
Source: PLoS One. 2025 Aug 8;20(8):e0329937. doi: 10.1371/journal.pone.0329937 (PMC12334010; doi:10.1371/journal.pone.0329937)
Supplement: S1 File — (DOCX) [file pone.0329937.s001.docx]

S1 File. Energy intensity change rate of Shimen

The 14th Five-Year Plan for National Economic and Social Development and the Long-term Vision Outline for 2035 in Shimen County reveal the energy consumption per ten thousand Yuan GDP of Shimen decreased by 28.5% between 2016 and 2020 [1]. We calculated Shimen County's annual average decrease rate in energy consumption per 10,000 yuan of GDP to be 6.5%.

Reference

1. Shimen County’s 14th Five-Year Plan for Economic and Social Development and the Long-Range Objectives through the Year 2035. [Cited May 6, 2024]

Available from: <https://www.shimen.gov.cn/group1/M00/01/FF/ClADFGSPxleAf-_oAD0YFYl1VtA290.pdf>
