# Supplementary material for: Scenario-based Kaya identity analysis for city-level carbon dioxide emissions
Source: PLoS One. 2025 Aug 8;20(8):e0329937. doi: 10.1371/journal.pone.0329937 (PMC12334010; doi:10.1371/journal.pone.0329937)
Supplement: S1 Table — (DOCX) [file pone.0329937.s002.docx]

|  | 2016 | 2017 | 2018 | 2019 | 2020 | 2021 | 2022 |
| --- | --- | --- | --- | --- | --- | --- | --- |
| Changning | 7.80% | 8.70% | 8.60% | 8.30% | 4.50% | 8.60% | 5.10% |
| Leiyang | 7.90% | 8.10% | 8.10% | 8.10% | 4.20% | 7.70% | 5.20% |
| Hengyang | 8.00% | 8.50% | 8.40% | 7.80% | 3.80% | 7.90% | 5.20% |
| Hengnan | 8.00% | 8.30% | 8.00% | 8.00% | 4.00% | 8.30% | 5.40% |
| Hengshan | 8.10% | 8.60% | 8.30% | 7.90% | 3.70% | 7.80% | 5.70% |
| Hengdong | 7.90% | 8.70% | 8.20% | 8.10% | 4.20% | 8.30% | 5.50% |
| Qidong | 8.20% | 8.90% | 8.80% | 8.20% | 4.30% | 8.40% | 5.00% |

S1 Table. The annual GDP change rate of seven county level administrative units of Hengyang from 2015 to 2022

Source [1-50]

Reference

1. Statistical Communiqué of Changning City on the 2016 National Economic and Social Development. [Cited April 12, 2024]. Available from: http://www.hnchangning.gov.cn/zwgk/szfbmxxgkml/tjj/ywl/20200210/i935617.html.
2. Statistical Communiqué of Changning City on the 2017 National Economic and Social Development. [Cited April 12, 2024]. Available from: http://www.hnchangning.gov.cn/zwgk/szfxxgkml/sjfb/sjfb/20200205/i600429.html.
3. Statistical Communiqué of Changning City on the 2018 National Economic and Social Development. [Cited April 21, 2023]. Available from: http://www.hnchangning.gov.cn/zwgk/szfxxgkml/sjfb/sjfb/20200205/i600335.html.
4. Statistical Communiqué of Changning City on the 2019 National Economic and Social Development. [ Cited April 12, 2023]. Available from: <http://www.hnchangning.gov.cn/zwgk/szfxxgkml/sjfb/sjfb/20200408/i1958697.html>.
5. Statistical Communiqué of Changning City on the 2020 National Economic and Social Development. [Cited April 15, 2024]. Available from: <https://www.hengyang.gov.cn/hystjj/tjgb/20210421/i2353439.html>.
6. Statistical Communiqué of Changning City on the 2021 National Economic and Social Development. [Cited April 21, 2023]. Available from: <http://www.hnchangning.gov.cn/zwgk/szfbmxxgkml/tjj/ywl/20220407/i2660108.html>.
7. Statistical Communiqué of Changning City on the 2022 National Economic and Social Development. [Cited April 21, 2023]. Available from: <http://www.hengyang.gov.cn/sjfb/tjgb/20230329/i2973111.html>.
8. Statistical Communiqué of Leiyang City on the 2016 National Economic and Social Development. [Cited May 3, 2025]. Available from: https://www.hengyang.gov.cn/hystjj/tjgb/20200201/i446082.html.
9. Statistical Communiqué of Leiyang City on the 2017 National Economic and Social Development. [Cited May 3, 2025]. Available from: https://www.leiyang.gov.cn/xxgk/szfxxgkml/sjfb/tjgb/20240424/i3322066.html.
10. Statistical Communiqué of Leiyang City on the 2018 National Economic and Social Development. [Cited May 3, 2025]. Available from: https://www.leiyang.gov.cn/xxgk/szfxxgkml/sjfb/tjgb/20240424/i3322058.html.
11. Statistical Communiqué of Leiyang City on the 2019 National Economic and Social Development. [Cited May 3, 2025]. Available from: https://www.leiyang.gov.cn/xxgk/szfxxgkml/sjfb/tjgb/20240424/i3322056.html.
12. Statistical Communiqué of Leiyang City on the 2020 National Economic and Social Development. [Cited May 3, 2025]. Available from: https://www.leiyang.gov.cn/xxgk/szfxxgkml/sjfb/tjgb/20240426/i3323505.html.
13. Statistical Communiqué of Leiyang City on the 2021 National Economic and Social Development. [Cited May 3, 2025]. Available from: https://www.leiyang.gov.cn/xxgk/szfxxgkml/sjfb/tjgb/20240424/i3322053.html.
14. Statistical Communiqué of Leiyang City on the 2022 National Economic and Social Development. [Cited May 3, 2025]. Available from: https://www.leiyang.gov.cn/xxgk/szfxxgkml/sjfb/tjgb/20240424/i3322052.html.
15. Statistical Communiqué of Hengyang County on the 2016 National Economic and Social Development. [Cited May 3, 2025]. Available from: http://www.hyx.gov.cn/hyxtjxxw/xxgk/ywgz/20200214/i1113335.html.
16. Statistical Communiqué of Hengyang County on the 2017 National Economic and Social Development. [Cited May 3, 2025]. Available from: http://www.hyx.gov.cn/zwgk/zfxxgkml/sjfb/20200214/i1042527.html.
17. Statistical Communiqué of Hengyang County on the 2018 National Economic and Social Development. [Cited May 3, 2025]. Available from: http://www.hyx.gov.cn/zwgk/zfxxgkml/sjfb/20200214/i1042515.html.
18. Statistical Communiqué of Hengyang County on the 2019 National Economic and Social Development. [Cited May 3, 2025]. Available from: https://www.hengyang.gov.cn/sjfb/tjgb/20200318/i1848927.html.
19. Statistical Communiqué of Hengyang County on the 2020 National Economic and Social Development. [Cited May 3, 2025]. Available from: https://www.hengyang.gov.cn/sjfb/tjgb/20210421/i2353381.html.
20. Statistical Communiqué of Hengyang County on the 2021 National Economic and Social Development. [Cited May 3, 2025]. Available from: https://www.hengyang.gov.cn/sjfb/tjgb/20220407/i2660162.html.
21. Statistical Communiqué of Hengyang County on the 2022 National Economic and Social Development. [Cited May 3, 2025]. Available from: https://www.hengyang.gov.cn/sjfb/tjgb/20230329/i2973093.html.
22. Statistical Communiqué of Hengnan County on the 2016 National Economic and Social Development. [Cited May 3, 2025]. Available from: https://www.hengnan.gov.cn/zwgk/sjfb/tjgb/20200207/i804005.html.
23. Statistical Communiqué of Hengnan County on the 2017 National Economic and Social Development. [Cited May 3, 2025]. Available from: https://www.hengnan.gov.cn/zwgk/sjfb/tjgb/20200207/i803998.html.
24. Statistical Communiqué of Hengnan County on the 2018 National Economic and Social Development. [Cited May 3, 2025]. Available from: https://www.hengnan.gov.cn/zwgk/sjfb/tjgb/20200207/i803994.html.
25. Statistical Communiqué of Hengnan County on the 2019 National Economic and Social Development. [Cited May 3, 2025]. Available from: https://www.hengnan.gov.cn/zwgk/zfgzbg/jjgzbg/20241111/i3502105.html.
26. Statistical Communiqué of Hengnan County on the 2020 National Economic and Social Development. [Cited May 3, 2025]. Available from: https://www.hengnan.gov.cn/zwgk/zfgzbg/jjgzbg/20241111/i3502103.html.
27. Statistical Communiqué of Hengnan County on the 2021 National Economic and Social Development. [Cited May 3, 2025]. Available from: https://www.hengnan.gov.cn/zwgk/sjfb/tjgb/20220407/i2660158.html.
28. Statistical Communiqué of Hengnan County on the 2022 National Economic and Social Development. [Cited May 3, 2025]. Available from: https://www.hengnan.gov.cn/zjhn/xqgk/jjgk/20230324/i2969590.html.
29. 2022 population data of Hengnan County. [Cited May 3, 2025]. Available from: <https://ceidata.cei.cn/>.
30. Statistical Communiqué of Hengshan County on the 2016 National Economic and Social Development. [Cited May 3, 2025]. Available from: https://www.hengshan.gov.cn/xzfgzbm/xtjj/fdzdgknr/sjfb/tjgb/20200605/i2052463.html.
31. Statistical Communiqué of Hengshan County on the 2017 National Economic and Social Development. [Cited May 3, 2025]. Available from: https://www.hengshan.gov.cn/xzfgzbm/xtjj/fdzdgknr/sjfb/tjgb/20200605/i2052462.html.
32. Statistical Communiqué of Hengshan County on the 2018 National Economic and Social Development. [Cited May 3, 2025]. Available from: https://www.hengshan.gov.cn/xzfgzbm/xtjj/fdzdgknr/sjfb/tjgb/20200605/i2052461.html.
33. Statistical Communiqué of Hengshan County on the 2019 National Economic and Social Development. [Cited May 3, 2025]. Available from: https://www.hengshan.gov.cn/xzfgzbm/xtjj/fdzdgknr/sjfb/tjgb/20200605/i2052460.html.
34. Statistical Communiqué of Hengshan County on the 2020 National Economic and Social Development. [Cited May 3, 2025]. Available from: https://www.hengshan.gov.cn/xzfgzbm/xtjj/fdzdgknr/sjfb/tjgb/20210428/i2359918.html.
35. Statistical Communiqué of Hengshan County on the 2021 National Economic and Social Development. [Cited May 3, 2025]. Available from: https://www.hengshan.gov.cn/xzfgzbm/xtjj/fdzdgknr/sjfb/tjgb/20220406/i2658660.html.
36. Statistical Communiqué of Hengshan County on the 2022 National Economic and Social Development. [Cited May 3, 2025]. Available from: https://www.hengshan.gov.cn/xzfgzbm/xtjj/fdzdgknr/sjfb/tjgb/20230307/i2944620.html.
37. Statistical Communiqué of Hengdong County on the 2016 National Economic and Social Development. [Cited May 3, 2025]. Available from: https://www.hengyang.gov.cn/sjfb/tjgb/20201012/i2167266.html.
38. Statistical Communiqué of Hengdong County on the 2017 National Economic and Social Development. [Cited May 3, 2025]. Available from: https://www.hengyang.gov.cn/hystjj/tjgb/20200201/i445985.html.
39. Statistical Communiqué of Hengdong County on the 2018 National Economic and Social Development. [Cited May 3, 2025]. Available from: https://www.hengyang.gov.cn/sjfb/tjgb/20200111/i63639.html.
40. Statistical Communiqué of Hengdong County on the 2019 National Economic and Social Development. [Cited May 3, 2025]. Available from: https://www.hengyang.gov.cn/sjfb/tjgb/20200318/i1848939.html.
41. Statistical Communiqué of Hengdong County on the 2020 National Economic and Social Development. [Cited May 3, 2025]. Available from: http://www.hengdong.gov.cn/zwgk/xzfxxgkml/tjsj/20210525/i2381127.html.
42. Statistical Communiqué of Hengdong County on the 2021 National Economic and Social Development. [Cited May 3, 2025]. Available from: https://www.hengyang.gov.cn/sjfb/tjgb/20220405/i2658056.html.
43. Statistical Communiqué of Hengdong County on the 2022 National Economic and Social Development. [Cited May 3, 2025]. Available from: https://www.hengyang.gov.cn/sjfb/tjgb/20230329/i2973102.html.
44. Statistical Communiqué of Qidong County on the 2016 National Economic and Social Development. [Cited May 3, 2025]. Available from: https://www.qdx.gov.cn/xxgk/sjfb/tjgb/20240415/i3313570.html.
45. Statistical Communiqué of Qidong County on the 2017 National Economic and Social Development. [Cited May 3, 2025]. Available from: https://www.qdx.gov.cn/xxgk/sjfb/tjgb/20240415/i3313574.html.
46. Statistical Communiqué of Qidong County on the 2018 National Economic and Social Development. [Cited May 3, 2025]. Available from: https://www.hengyang.gov.cn/sjfb/tjgb/20200111/i63633.html
47. Statistical Communiqué of Qidong County on the 2019 National Economic and Social Development. [Cited May 3, 2025]. Available from: https://www.qdx.gov.cn/xxgk/tjsj/20200604/i2046417.html.
48. Statistical Communiqué of Qidong County on the 2020 National Economic and Social Development. [Cited May 3, 2025]. Available from: https://www.qdx.gov.cn/zjqd/qdgk/gmjjhshfz/20210622/i2410086.html.
49. Statistical Communiqué of Qidong County on the 2021 National Economic and Social Development. [Cited May 3, 2025]. Available from: https://www.qdx.gov.cn/zjqd/qdgk/gmjjhshfz/20220601/i2704897.html.
50. Statistical Communiqué of Qidong County on the 2022 National Economic and Social Development. [Cited May 3, 2025]. Available from: https://www.qdx.gov.cn/xxgk/sjfb/tjgb/20240415/i3313543.html.
