# Supplementary material for: Scenario-based Kaya identity analysis for city-level carbon dioxide emissions
Source: PLoS One. 2025 Aug 8;20(8):e0329937. doi: 10.1371/journal.pone.0329937 (PMC12334010; doi:10.1371/journal.pone.0329937)
Supplement: S3 Table — (DOCX) [file pone.0329937.s004.docx]

S3 Table. The annual population change rate of seven county level administrative units of Hengyang from 2015 to 2022

|  | 2015 | 2016 | 2017 | 2018 | 2019 | 2021 | 2022 |
| --- | --- | --- | --- | --- | --- | --- | --- |
| Changning | 0.70% | -0.36% | -0.94% | -2.26% | -0.94% | -0.39% | -0.19% |
| Leiyang | 0.68% | -0.93% | -1.69% | -2.13% | -1.47% | 1.69% | -0.54% |
| Hengyang | -0.22% | -2.58% | -2.00% | -2.15% | -1.09% | -0.59% | -0.49% |
| Hengnan | 0.58% | -0.82% | -2.59% | -2.03% | -1.20% | 0.23% | -0.64% |
| Hengshan | 0.46% | -1.02% | -2.13% | -2.78% | -1.30% | -0.48% | -0.66% |
| Hengdong | 0.19% | -0.78% | -1.41% | -2.23% | -0.94% | -0.94% | -0.71% |
| Qidong | 0.38% | -0.43% | -0.98% | -1.92% | -1.38% | 0.17% | -0.70% |

Source [1-66]

Notes: The annual population change rates in 2020 was not used. The reasons were explained in the main text. The population change rate includes data in 2015, so the sample sizes for population change rate and GDP growth rate are the same.

Reference

1. Statistical Communiqué of Changning City on the 2014 National Economic and Social Development. [Cited May 19, 2025]. Available from: https://www.hengyang.gov.cn/hystjj/tjgb/20200201/i446277.html.
2. Statistical Communiqué of Changning City on the 2015 National Economic and Social Development. [Cited April 12, 2024]. Available from: http://www.hnchangning.gov.cn/zjcn/cngk/jjgs/20200205/i572125.html. .
3. Statistical Communiqué of Changning City on the 2016 National Economic and Social Development. [Cited April 12, 2024]. Available from: http://www.hnchangning.gov.cn/zwgk/szfbmxxgkml/tjj/ywl/20200210/i935617.html.
4. Statistical Communiqué of Changning City on the 2017 National Economic and Social Development. [Cited April 12, 2024]. Available from: http://www.hnchangning.gov.cn/zwgk/szfxxgkml/sjfb/sjfb/20200205/i600429.html.
5. Statistical Communiqué of Changning City on the 2018 National Economic and Social Development. [Cited April 21, 2023]. Available from: http://www.hnchangning.gov.cn/zwgk/szfxxgkml/sjfb/sjfb/20200205/i600335.html.
6. Statistical Communiqué of Changning City on the 2019 National Economic and Social Development. [ Cited April 12, 2023]. Available from: <http://www.hnchangning.gov.cn/zwgk/szfxxgkml/sjfb/sjfb/20200408/i1958697.html>.
7. Statistical Communiqué of Changning City on the 2020 National Economic and Social Development. [Cited April 15, 2024]. Available from: <https://www.hengyang.gov.cn/hystjj/tjgb/20210421/i2353439.html>.
8. Statistical Communiqué of Changning City on the 2021 National Economic and Social Development. [Cited April 21, 2023]. Available from: <http://www.hnchangning.gov.cn/zwgk/szfbmxxgkml/tjj/ywl/20220407/i2660108.html>. .
9. Statistical Communiqué of Changning City on the 2022 National Economic and Social Development. [Cited April 21, 2023]. Available from: <http://www.hengyang.gov.cn/sjfb/tjgb/20230329/i2973111.html>.
10. Statistical Communiqué of Leiyang City on the 2014 National Economic and Social Development. [Cited May 19, 2025]. Available from: https://www.hengyang.gov.cn/sjfb/tjgb/20201012/i2167440.html.
11. Statistical Communiqué of Leiyang City on the 2015 National Economic and Social Development. [Cited May 3, 2025]. Available from: <https://www.hengyang.gov.cn/sjfb/tjgb/20201012/i2167314.html>.
12. Statistical Communiqué of Leiyang City on the 2016 National Economic and Social Development. [Cited May 3, 2025]. Available from:https://www.hengyang.gov.cn/hystjj/tjgb/20200201/i446082.html.
13. Statistical Communiqué of Leiyang City on the 2017 National Economic and Social Development. [Cited May 3, 2025]. Available from: https://www.leiyang.gov.cn/xxgk/szfxxgkml/sjfb/tjgb/20240424/i3322066.html.
14. Statistical Communiqué of Leiyang City on the 2018 National Economic and Social Development. [Cited May 3, 2025]. Available from: https://www.leiyang.gov.cn/xxgk/szfxxgkml/sjfb/tjgb/20240424/i3322058.html.
15. Statistical Communiqué of Leiyang City on the 2019 National Economic and Social Development. [Cited May 3, 2025]. Available from: https://www.leiyang.gov.cn/xxgk/szfxxgkml/sjfb/tjgb/20240424/i3322056.html.
16. Statistical Communiqué of Leiyang City on the 2020 National Economic and Social Development. [Cited May 3, 2025]. Available from: https://www.leiyang.gov.cn/xxgk/szfxxgkml/sjfb/tjgb/20240426/i3323505.html.
17. Statistical Communiqué of Leiyang City on the 2021 National Economic and Social Development. [Cited May 3, 2025]. Available from: https://www.leiyang.gov.cn/xxgk/szfxxgkml/sjfb/tjgb/20240424/i3322053.html.
18. Statistical Communiqué of Leiyang City on the 2022 National Economic and Social Development. [Cited May 3, 2025]. Available from: https://www.leiyang.gov.cn/xxgk/szfxxgkml/sjfb/tjgb/20240424/i3322052.html.
19. Statistical Communiqué of Hengyang County on the 2014 National Economic and Social Development. [Cited May 3, 2025]. Available from: http://www.hyx.gov.cn/hyxtjxxw/xxgk/xxgkndbg/20200214/i1113294.html. Accessed May 20, 2025.
20. Statistical Communiqué of Hengyang County on the 2015 National Economic and Social Development. [Cited May 3, 2025]. Available from: https://www.hengyang.gov.cn/hystjj/tjgb/20200201/i446212.html.
21. Statistical Communiqué of Hengyang County on the 2016 National Economic and Social Development. [Cited May 3, 2025]. Available from: http://www.hyx.gov.cn/hyxtjxxw/xxgk/ywgz/20200214/i1113335.html.
22. Statistical Communiqué of Hengyang County on the 2017 National Economic and Social Development. [Cited May 3, 2025]. Available from: http://www.hyx.gov.cn/zwgk/zfxxgkml/sjfb/20200214/i1042527.html.
23. Statistical Communiqué of Hengyang County on the 2018 National Economic and Social Development. [Cited May 3, 2025]. Available from: http://www.hyx.gov.cn/zwgk/zfxxgkml/sjfb/20200214/i1042515.html.
24. Statistical Communiqué of Hengyang County on the 2019 National Economic and Social Development. [Cited May 3, 2025]. Available from: https://www.hengyang.gov.cn/sjfb/tjgb/20200318/i1848927.html.
25. Statistical Communiqué of Hengyang County on the 2020 National Economic and Social Development. [Cited May 3, 2025]. Available from: https://www.hengyang.gov.cn/sjfb/tjgb/20210421/i2353381.html.
26. Statistical Communiqué of Hengyang County on the 2021 National Economic and Social Development. [Cited May 3, 2025]. Available from: https://www.hengyang.gov.cn/sjfb/tjgb/20220407/i2660162.html.
27. Statistical Communiqué of Hengyang County on the 2022 National Economic and Social Development. [Cited May 3, 2025]. Available from: https://www.hengyang.gov.cn/sjfb/tjgb/20230329/i2973093.html.
28. Statistical Communiqué of Hengnan County on the 2014 National Economic and Social Development. [Cited May 20, 2025]. Available from: https://www.hengnan.gov.cn/zwgk/sjfb/tjgb/20200207/i804036.html.
29. Statistical Communiqué of Hengnan County on the 2015 National Economic and Social Development. [Cited May 3, 2025]. Available from: https://www.hengnan.gov.cn/zwgk/sjfb/tjgb/20200207/i804012.html.
30. Statistical Communiqué of Hengnan County on the 2016 National Economic and Social Development. [Cited May 3, 2025]. Available from: https://www.hengnan.gov.cn/zwgk/sjfb/tjgb/20200207/i804005.html.
31. Statistical Communiqué of Hengnan County on the 2017 National Economic and Social Development. [Cited May 3, 2025]. Available from: https://www.hengnan.gov.cn/zwgk/sjfb/tjgb/20200207/i803998.html.
32. Statistical Communiqué of Hengnan County on the 2018 National Economic and Social Development. [Cited May 3, 2025]. Available from: https://www.hengnan.gov.cn/zwgk/sjfb/tjgb/20200207/i803994.html.
33. Statistical Communiqué of Hengnan County on the 2019 National Economic and Social Development. [Cited May 3, 2025]. Available from: https://www.hengnan.gov.cn/zwgk/zfgzbg/jjgzbg/20241111/i3502105.html.
34. Statistical Communiqué of Hengnan County on the 2020 National Economic and Social Development. [Cited May 3, 2025]. Available from: https://www.hengnan.gov.cn/zwgk/zfgzbg/jjgzbg/20241111/i3502103.html.
35. Statistical Communiqué of Hengnan County on the 2021 National Economic and Social Development. [Cited May 3, 2025]. Available from: https://www.hengnan.gov.cn/zwgk/sjfb/tjgb/20220407/i2660158.html.
36. Statistical Communiqué of Hengnan County on the 2022 National Economic and Social Development. [Cited May 3, 2025]. Available from: https://www.hengnan.gov.cn/zjhn/xqgk/jjgk/20230324/i2969590.html.
37. 2022 population data of Hengnan County. Available from: <https://ceidata.cei.cn/>
38. Statistical Communiqué of Hengshan County on the 2014 National Economic and Social Development. [Cited May 20, 2025]. Available from: https://www.hengshan.gov.cn/xzfgzbm/xtjj/fdzdgknr/sjfb/tjgb/20200605/i2052465.html.
39. Statistical Communiqué of Hengshan County on the 2015 National Economic and Social Development. [Cited May 3, 2025]. Available from: https://www.hengshan.gov.cn/xzfgzbm/xtjj/fdzdgknr/sjfb/tjgb/20200605/i2052464.html.
40. Statistical Communiqué of Hengshan County on the 2016 National Economic and Social Development. [Cited May 3, 2025]. Available from: https://www.hengshan.gov.cn/xzfgzbm/xtjj/fdzdgknr/sjfb/tjgb/20200605/i2052463.html.
41. Statistical Communiqué of Hengshan County on the 2017 National Economic and Social Development. [Cited May 3, 2025]. Available from: https://www.hengshan.gov.cn/xzfgzbm/xtjj/fdzdgknr/sjfb/tjgb/20200605/i2052462.html.
42. Statistical Communiqué of Hengshan County on the 2018 National Economic and Social Development. [Cited May 3, 2025]. Available from: https://www.hengshan.gov.cn/xzfgzbm/xtjj/fdzdgknr/sjfb/tjgb/20200605/i2052461.html.
43. Statistical Communiqué of Hengshan County on the 2019 National Economic and Social Development. [Cited May 3, 2025]. Available from: https://www.hengshan.gov.cn/xzfgzbm/xtjj/fdzdgknr/sjfb/tjgb/20200605/i2052460.html.
44. Statistical Communiqué of Hengshan County on the 2020 National Economic and Social Development. [Cited May 3, 2025]. Available from: https://www.hengshan.gov.cn/xzfgzbm/xtjj/fdzdgknr/sjfb/tjgb/20210428/i2359918.html.
45. Statistical Communiqué of Hengshan County on the 2021 National Economic and Social Development. [Cited May 3, 2025]. Available from: https://www.hengshan.gov.cn/xzfgzbm/xtjj/fdzdgknr/sjfb/tjgb/20220406/i2658660.html.
46. Statistical Communiqué of Hengshan County on the 2022 National Economic and Social Development. [Cited May 3, 2025]. Available from: https://www.hengshan.gov.cn/xzfgzbm/xtjj/fdzdgknr/sjfb/tjgb/20230307/i2944620.html.
47. The population data situation of Hengndong County in 2014. [Cited May 20, 2025]. Available from: https://ceidata.cei.cn/.
48. Statistical Communiqué of Hengdong County on the 2015 National Economic and Social Development. [Cited May 3, 2025]. Available from: https://www.hengyang.gov.cn/sjfb/tjgb/20201012/i2167317.html.
49. Statistical Communiqué of Hengdong County on the 2016 National Economic and Social Development. [Cited May 3, 2025]. Available from: https://www.hengyang.gov.cn/sjfb/tjgb/20201012/i2167266.html.
50. Statistical Communiqué of Hengdong County on the 2017 National Economic and Social Development. [Cited May 3, 2025]. Available from: https://www.hengyang.gov.cn/hystjj/tjgb/20200201/i445985.html.
51. Statistical Communiqué of Hengdong County on the 2018 National Economic and Social Development. [Cited May 3, 2025]. Available from: https://www.hengyang.gov.cn/sjfb/tjgb/20200111/i63639.html.
52. Statistical Communiqué of Hengdong County on the 2019 National Economic and Social Development. [Cited May 3, 2025]. Available from: https://www.hengyang.gov.cn/sjfb/tjgb/20200318/i1848939.html.
53. Statistical Communiqué of Hengdong County on the 2020 National Economic and Social Development. [Cited May 3, 2025]. Available from: http://www.hengdong.gov.cn/zwgk/xzfxxgkml/tjsj/20210525/i2381127.html.
54. Statistical Communiqué of Hengdong County on the 2021 National Economic and Social Development. [Cited May 3, 2025]. Available from: https://www.hengyang.gov.cn/sjfb/tjgb/20220405/i2658056.html.
55. Statistical Communiqué of Hengdong County on the 2022 National Economic and Social Development. [Cited May 3, 2025]. Available from: https://www.hengyang.gov.cn/sjfb/tjgb/20230329/i2973102.html.
56. The permanent population of Qidong County in 2014. <https://ceidata.cei.cn/.> Accessed May 20, 2025.
57. Statistical Communiqué of Qidong County on the 2015 National Economic and Social Development. [Cited May 3, 2025]. Available from: https://www.qdx.gov.cn/xxgk/xxgkml/xzfgzbmxxgkml/xtjj/tzgg/20250114/i3557440.html.
58. The permanent population of Qidong County from 1999 to 2023. [Cited May 3, 2025]. [https://population.gotohui.com/show-26539.](https://ceidata.cei.cn/.)
59. Statistical Communiqué of Qidong County on the 2016 National Economic and Social Development. [Cited May 3, 2025]. Available from: https://www.qdx.gov.cn/xxgk/sjfb/tjgb/20240415/i3313570.html.
60. Statistical Communiqué of Qidong County on the 2017 National Economic and Social Development. [Cited May 3, 2025]. Available from: https://www.qdx.gov.cn/xxgk/sjfb/tjgb/20240415/i3313574.html.
61. Statistical Communiqué of Qidong County on the 2018 National Economic and Social Development. [Cited May 3, 2025]. Available from: https://www.hengyang.gov.cn/sjfb/tjgb/20200111/i63633.html
62. Statistical Communiqué of Qidong County on the 2019 National Economic and Social Development. [Cited May 3, 2025]. Available from: https://www.qdx.gov.cn/xxgk/tjsj/20200604/i2046417.html.
63. Statistical Communiqué of Qidong County on the 2020 National Economic and Social Development. [Cited May 3, 2025]. Available from: https://www.qdx.gov.cn/zjqd/qdgk/gmjjhshfz/20210622/i2410086.html.
64. Statistical Communiqué of Qidong County on the 2021 National Economic and Social Development. [Cited May 3, 2025]. Available from: <https://www.qdx.gov.cn/zjqd/qdgk/gmjjhshfz/20220601/i2704897.html>.
65. Statistical Communiqué of Qidong County on the 2022 National Economic and Social Development. [Cited May 3, 2025]. Available from: <https://www.qdx.gov.cn/xxgk/sjfb/tjgb/20240415/i3313543.html>.
66. Hengyang City Seventh National Population Census Bulletin [1] (No. 1) — Population Status of the Entire City and Its Counties, Districts, and Municipal Areas. [Cited May 28, 2025]. Available at: https://www.hengyang.gov.cn/sjfb/tjgb/20210616/i2401884.html.
