# Supplementary material for: Scenario-based Kaya identity analysis for city-level carbon dioxide emissions
Source: PLoS One. 2025 Aug 8;20(8):e0329937. doi: 10.1371/journal.pone.0329937 (PMC12334010; doi:10.1371/journal.pone.0329937)
Supplement: S4 Table — (DOCX) [file pone.0329937.s005.docx]

S4 Table. The annual average energy intensity change rate of twenty county level administrative units in Hunan province from 2015 to 2020

| County / County level City | Annual average energy intensity change rate during the 13^th^ five year period (2016-2020) |
| --- | --- |
| Changning | -4.80% |
| Leiyang | -3.60% |
| Hengdong | -2.30% |
| Shimen | -6.50% |
| Jingshi | -7.80% |
| Taoyuan | -13.40% |
| Guiyang | -5.40% |
| Ningyuan | -5.30% |
| Zhijiang | -4.00% |
| Jingzhou | -5.05% |
| Shuangfeng | -3.00% |
| Lengshuijiang | -4.70% |
| Huayuan | -7.30% |
| Jianghua | -8.30% |
| Shaoyang | -6.70% |
| Cili | -3.43% |
| Yongshun | -2.00% |
| Changsha | -3.70% |
| Yizhang | -4.00% |
| Xinshao | -7.70% |

Source [1-22]

Reference

1. Shimen County’s 14th Five-Year Plan for Economic and Social Development and the Long-Range Objectives through the Year 2035. [Cited May 6, 2024]

Available from: <https://www.shimen.gov.cn/group1/M00/01/FF/ClADFGSPxleAf-_oAD0YFYl1VtA290.pdf>.

1. Changning City’s 14th Five-Year Plan for Economic and Social Development and the Long-Range Objectives through the Year 2035. [Cited May 1, 2023]. Available from: <http://www.hnchangning.gov.cn/DFS/file/2022/11/21/2022112114321846897l3vv.pdf>.
2. Leiyang City’s14th Five-Year Plan for Economic and Social Development and the Long-Range Objectives through the Year 2035. [Cited May 1, 2023]. Available from: https://www.leiyang.gov.cn/xxgk/szfxxgkml/ghjh/20240312/i3277519.html.
3. Hengdong County’s 14th Five-Year Plan for Economic and Social Development and the Long-Range Objectives through the Year 2035. [Cited May 1, 2023]. Available from: http://www.hengdong.gov.cn/ztzx/hdsswghgyzl/20220308/i2636412.html.
4. Guiyang County’s 14th Five-Year Plan for Economic and Social Development and the Long-Range Objectives through the Year 2035. [Cited May 27, 2025]. Available from: <http://www.czswdx.cn/162/5111.html>.
5. Ninyuan County’s 14th Five-Year Plan for Economic and Social Development and the Long-Range Objectives through the Year 2035. Available from: https://www.nyx.gov.cn/nyx/ghqw/202111/a509768f079d4586a1305972f4af6176/files/e9d2d737f6e64aca98e51e3f26f0882c.pdf.
6. Taoyuan County’s 14th Five-Year Plan for Economic and Social Development and the Long-Range Objectives through the Year 2035. [Cited May 27, 2025]. Available from: https://www.taoyuan.gov.cn/zwgk/public/6616339/1133195461.html.
7. Jingshi City’s 14th Five-Year Plan for Economic and Social Development and the Long-Range Objectives through the Year 2035. [Cited May 28, 2025]. Available from: https://www.jinshishi.gov.cn/zwgk/public/6616338/1183527271.html.
8. Zhijiang Dong Autonomous County’s 14th Five-Year Plan for Economic and Social Development and the Long-Range Objectives through the Year 2035. [Cited May 28, 2025]. Available from: <http://www.chnzj.gov.cn/chnzj/c132843/202111/754d65786d1945178ac514621cdf6e65.shtml>.
9. Jingzhou Miao and Dong Autonomous County’s 14th Five-Year Plan for Economic and Social Development and the Long-Range Objectives through the Year 2035. [Cited May 28, 2025]. Available from: <http://www.jzx.gov.cn/jzx/c132837/202111/1042129cbd214f2bb3817c313fda136d.shtml>.
10. Shuangfeng County’s 14th Five-Year Plan for Economic and Social Development and the Long-Range Objectives through the Year 2035. [Cited May 28, 2025]. Available from: https://www.hnsf.gov.cn/hnsf/tzgg14/202111/4e48e825bca44c2ca20a086a0488dd04/files/f8d87af534b84a8f8f56e4e52226401f.pdf.
11. Lengshuijiang City’s 14th Five-Year Plan for Economic and Social Development and the Long-Range Objectives through the Year 2035. [Cited May 28, 2025]. Available from: https://www.lsj.gov.cn/lsj/zwgk/bmxxgkml/szfgzbm/sfzhggj/ghjh10/202107/2063e63b673a4c6c9958afcc427f505b/files/995e4b68ee1b40fbad5e3f3f8211a3b8.pdf.
12. Huayuan County’s 14th Five-Year Plan for Economic and Social Development and the Long-Range Objectives through the Year 2035. [Cited May 29, 2025]. Available from: http://www.huayuan.gov.cn/zwgk_23240/xzfxxgkml_23243/ghjh_23255/202207/t20220721_1914494.html.
13. Jianghua Yao Autonomous County’s 14th Five-Year Plan for Economic and Social Development and the Long-Range Objectives through the Year 2035. [Cited May 29, 2025]. Available from: <http://www.jh.gov.cn/jh/toutiao/202201/18137b9a707041d0a4c425cefcd50412/files/19147a8edcdf476dbfcd774a3f9102d9.pdf>.
14. Jianghua Yao Autonomous County’s 13th Five-Year Plan for Economic and Social Development. [Cited May 29, 2025]. Available from: http://www.jh.gov.cn/jh/lsgh/202110/6ab8177a067e491e9e3fbfbec5c9a175.shtml.
15. Shaoyang County’s 14th Five-Year Plan for Economic and Social Development and the Long-Range Objectives through the Year 2035. [Cited May 29, 2025]. Available from: https://www.syx.gov.cn/syx/dzs/ebook.shtml?#page/20.
16. Cili County’s 14th Five-Year Plan for Economic and Social Development and the Long-Range Objectives through the Year 2035. [Cited May 29, 2025]. Available from: <https://www.cili.gov.cn/c3395/20210928/i625393.html>.
17. Yongshun County’s 14th Five-Year Plan for Economic and Social Development and the Long-Range Objectives through the Year 2035. [Cited May 29, 2025]. Available from: http://www.ysx.gov.cn/zwgk/xzfxxgkml/ghjh/202405/P020240514391013581945.pdf.
18. Changsha County’s 14th Five-Year Plan for Economic and Social Development and the Long-Range Objectives through the Year 2035. [Cited May 29, 2025]. Available from: http://www.csx.gov.cn/zwgk/zfxxgkml/fdzdgknr/ghxx_2020/gmjjhshfzgh_2020/202112/P020220118566616799437.pdf.
19. Yizhang County’s 14th Five-Year Plan for Economic and Social Development and the Long-Range Objectives through the Year 2035. [Cited May 27, 2025]. Available from: http://www.app.czs.gov.cn/yizhang/2/28/55746/content_3350477.html.
20. Xinshao County’s 14th Five-Year Plan for Economic and Social Development and the Long-Range Objectives through the Year 2035. [Cited May 27, 2025]. Available from: <https://www.xinshao.gov.cn/xinshao/xshfz/202302/2e25be9e389d40418bff9c7da0208183.shtml>.
21. Xinshao County’s 13th Five-Year Plan for Economic and Social Development. [Cited May 27, 2025]. Available from: <https://www.xinshao.gov.cn/xinshao/s3w01/2020listnr.shtml>.
